# Population connectivity and genetic offset in the spawning coral *Acropora digitifera* in Western Australia

**DOI:** 10.1111/mec.16498

**Published:** 2022-06-05

**Authors:** Arne A. S. Adam, Luke Thomas, Jim Underwood, James Gilmour, Zoe T. Richards

**Affiliations:** ^1^ Coral Conservation and Research Group, Trace and Environmental DNA Laboratory, School of Molecular and Life Sciences Curtin University Bentley Western Australia; ^2^ Australian Institute of Marine Science IOMRC, The University of Western Australia Crawley Western Australia; ^3^ The UWA Oceans Institute, Oceans Graduate School The University of Western Australia Crawley Western Australia; ^4^ Collections and Research Western Australian Museum Welshpool Western Australia

**Keywords:** broadcast corals, climate change, gene–environmental associations, North‐west Australia, population genetics

## Abstract

Anthropogenic climate change has caused widespread loss of species biodiversity and ecosystem productivity across the globe, particularly on tropical coral reefs. Predicting the future vulnerability of reef‐building corals, the foundation species of coral reef ecosystems, is crucial for cost‐effective conservation planning in the Anthropocene. In this study, we combine regional population genetic connectivity and seascape analyses to explore patterns of *genetic offset* (the mismatch of gene–environmental associations under future climate conditions) in *Acropora digitifera* across 12 degrees of latitude in Western Australia. Our data revealed a pattern of restricted gene flow and limited genetic connectivity among geographically distant reef systems. Environmental association analyses identified a suite of loci strongly associated with the regional temperature variation. These loci helped forecast future genetic offset in gradient forest and generalized dissimilarity models. These analyses predicted pronounced differences in the response of different reef systems in Western Australia to rising temperatures. Under the most optimistic future warming scenario (RCP 2.6), we predicted a general pattern of increasing genetic offset with latitude. Under the extreme climate scenario (RCP 8.5 in 2090–2100), coral populations at the Ningaloo World Heritage Area were predicted to experience a higher mismatch between current allele frequencies and those required to cope with local environmental change, compared to populations in the inshore Kimberley region. The study suggests complex and spatially heterogeneous patterns of climate‐change vulnerability in coral populations across Western Australia, reinforcing the notion that regionally tailored conservation efforts will be most effective at managing coral reef resilience into the future.

## 1 INTRODUCTION

The impacts of climate change are intensifying across ecosystems on multiple levels (Malhi et al., 2020), affecting not only species demography and dispersal, both of which underlie short term recovery, but also the genetic diversity and metapopulation structure that determine longer‐term recovery and adaptation (Osman et al., 2018; Pauls et al., 2013). Recurrent disturbances affect reproductive output (Hughes et al., 2019), the strength of connectivity networks and threaten to erode population resilience (Thomas et al., 2017, 2020) which could lead to local extinction events (Hoffmann & Sgro, 2011; Matz et al., 2020; Richards et al., 2021) and jeopardize ecosystem functioning (Benkwitt et al., 2020; Dietzel et al., 2021). In marine systems, monitoring changes in connectivity and genetic diversity among local populations at different spatio‐temporal scales are central to assessing their vulnerability to a warming planet (Kleypas et al., 2016; Oscar, 2017; Veron et al., 2009). For this reason, it is critical to integrate genetic data into conservation planning and protected area management (Gaitán‐Espitia & Hobday, 2021; Underwood et al., 2013).

A complex array of environmental and biological processes influence marine metapopulations (Guan et al., 2020; Suggett & Smith, 2020), so it can be difficult to extrapolate connectivity patterns from genetic variation (Oscar, 2017). Seascape genomic studies seek to investigate how (a)biotic factors such as environmental and biological parameters, as well as demographic processes are associated with genetic variation to identify potential drivers of population structure in the marine realm (Balkenhol et al., 2017; Riginos et al., 2016; Selmoni et al., 2020). Seascape analyses have revealed the role of the environment in shaping patterns of larval dispersal and coral population connectivity (Riginos et al., 2016; Riginos & Liggins, 2013; Selkoe et al., 2016; Thomas et al., 2015; Treml et al., 2012; Underwood et al., 2020, 2009, 2013). Gene–environment association analyses (GEAs) provide a means to explore the influence of the physical environment on the genetic structure of populations (Duruz et al., 2019; Rellstab et al., 2015; Selmoni et al., 2020). Additionally, random forest (gradient forest [GF]) and generalized dissimilarity models (GDM) of individual single nucleotide polymorphisms (SNPs) with environmental variables are valuable tools to investigate the adaptive capacity at broader spatial and temporal scales by evaluating the goodness of fit for the response of specific variant sites (in this case, SNPs) to specific environmental conditions (Fitzpatrick & Keller, 2015). Good performing models are then used to estimate spatial variation in the existing GEAs, and to determine if present‐day GEAs can be maintained under changing climate conditions (Fitzpatrick & Keller, 2015).

Genetic offset (Fitzpatrick & Keller, 2015) is the difference in the genetic composition of a population under present‐day versus projected future climate conditions. Therefore, estimates of genetic offset can be used to evaluate the level of allelic shift or adaptation required to avoid disrupting present‐day gene–environmental relationships (Fitzpatrick & Keller, 2015). Based on this analysis, a large genetic offset could lead to the reduced likelihood that a given population can adapt rapidly enough to survive future climate conditions. Several studies have investigated the potential link between environmental conditions and loci under selection in coral populations, using genetic markers and environmental parameters for coral growth and survival, such as tidal height, sea surface temperature and water clarity (Selmoni et al., 2020; Underwood et al., 2020, 2018). However, few studies have integrated GEAs to predict the local adaptive potential over time (Bay et al., 2017; Wood et al., 2021), population connectivity beyond the study area (Selmoni et al., 2020), or to examine how these associations affect the species' genetic composition and the adaptive potential of populations more generally (Gervais et al., 2021).

In Western Australia, large‐scale population connectivity studies have combined genotype data with environmental variables into integrated seascape analyses (Thomas et al., 2017, 2020; Underwood, 2009; Underwood et al., 2017, 2020, 2018, 2007, 2006, 2013). However, no study has explored how genetic structure patterns in these populations translate to climate change vulnerability. Here, we explore patterns of genetic offset in the ubiquitous broadcast spawning coral, *Acropora digitifera* Dana 1846, across north Western Australia by combining genotyping by sequencing (GBS) data with random forest and generalized dissimilarity models. First, we used the GBS approach to measure levels of spatial genetic structure across Western Australia to infer levels of reproductive isolation within and among separate geographic populations. Secondly, we utilized gene–environment association analyses to identify putatively adaptive variants likely to be under directional selection. Finally, we used these loci to predict mismatches in GEAs under future climate scenarios.

## 2 MATERIALS AND METHODS

### 2.1 Sample collection and genotype‐by‐sequencing filtering

**FIGURE 1:**
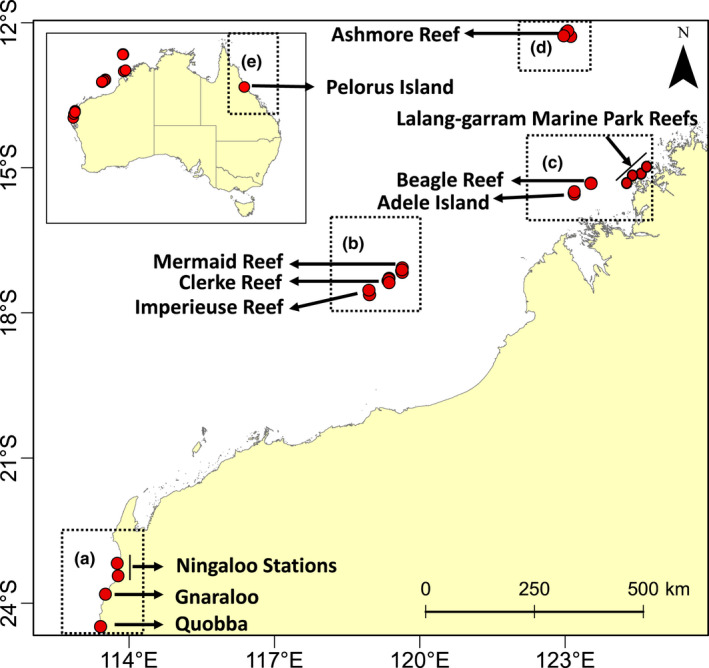
Map showing the 32 sites (red circles) sampled across five reef systems; (a) Ningaloo Coast World Heritage Area, (b) Rowley Shoals, (c) inshore Kimberley, (d) Ashmore Reef, (e) Pelorus Island, GBR

Population samples were collected from five reef systems (Figure 1): (1) The oceanic reef systems of Ashmore Reef and (2) the Rowley Shoals; (3) the turbid and macro‐tidal inshore Kimberley reef system (Adele Island, Beagle Reef and the nearshore fringing reefs within the Lalang‐garram Marine Park); (4) the fringing reefs of Gnaraloo, Quobba and Ningaloo Stations within the Ningaloo Coast World Heritage Area; and (5) Pelorus Island, mid‐shelf central Great Barrier Reef (GBR). GBR samples were included to provide broad evolutionary and geographic context to the levels of diversity and divergence detected among reef systems in Western Australia. A total of 756 *A. digitifera* samples (~1–6 cm^3^) were collected from 31 sites across the four aforementioned reef systems in Western Australia (Figure 1 and Table S1), along with an additional 33 samples collected from Pelorus Island (GBR). Samples were identified in the field according to the morphological description provided by Wallace (1999). Samples were stored in 100% ethanol, subsampled and sent to Diversity Array Technology Pty Ltd (DArT P/L) for DNA extraction, library preparation, sequencing and SNP calling using the same protocol as in Thomas et al. (2020). Furthermore, sequencing tags were blasted against the available *Acropora digitifera* genome (Shinzato et al., 2011) to confirm they belonged to the coral host and not the symbiont. Before quality control filtering (QC), raw loci sequences, averaging 1,283,302 (±151,230 SD) reads per sample (Table 1), were aligned to available *Symbiodinium* symbiont genomes (Aranda et al., 2016; Lin et al., 2015; Liu et al., 2018; Shoguchi et al., 2018, 2013) and any sequences with a *blastn* e‐value below 10^−3^ were discarded. Furthermore, a Euclidean distance matrix was generated based on replicate genotype data for a subset of samples. Only unique multilocus genotypes with a distance greater than the hamming distance between the replicates of individuals were retained, while others were considered potential clones and were removed from analysis.

**TABLE 1:** Generic and genetic metrics

Species	*N*	*N*_G_	N after QC	Reads/sample	SNPs	SNPs after *Symb* removal	QC	Neutral	Outlier	*F*_ST_	*H*_T_	*A*_R_
*Acropora digitifera*	789	787	704	1,283,302 (± 151,230 SD)	38,456	38,408	1550	1193	339	0.062	0.365	1.334

*Notes*: Total number of samples collected (*N*), Total number of unique genotypes after clone removal (*N*_G_), total number of genotypes after quality control check (individual and locus call rate >0.7), average number of reads per sample (±SD), number of single nucleotide polymorphisms (SNPs) after DArTsoft‐14 pipeline, SNPs after symbiont annotations, number of SNPs after QC, number of neutral and outlier loci after using BayeScan, overall *F*_ST_, expected heterozygosity (*H*_T_) and allelic richness (*A_R_*).

Initial screening of the DArT SNP data identified all individuals from the Lalang‐garram Marine Park reefs in the inshore Kimberley region (Jackson Island, Haywood Island, Augustus Island and Okenia Island) as outliers, probably representing a cryptic species (Tables S2 and S3, Figure S1). These samples were excluded from downstream analyses. After excluding the Lalang‐Garram Marine Park sample data, the remaining dataset returned 38,456 single nucleotide polymorphisms (SNPs) (Table 1) with a mean coverage of 36.15 (±0.155 SE) across the variant sites (min coverage: 5, max coverage: 243). In total, 48 loci aligned with *Symbiodinidae* sequences with an e‐value below the threshold and were discarded for downstream filtering (Table 1). Two genotypes, one from Adele Island site 1 and one from Rowley Shoals Clerke Reef site C11, were characterized as clones based on the hamming distance in the Euclidean distance matrix of replicates and were removed. The remaining dataset of 38,408 loci across five reef systems from 28 different sites (Table 1, Table S1) was filtered for call rate (>0.70) across loci and individuals (Figures S2a and b), average repeatability of alleles for every locus (>0.70), minor allele frequency (>0.05), sequencing coverage (>10×) and Hardy–Weinberg equilibrium (Thomas et al., 2020). Furthermore, secondary SNPs located within the same fragment were removed as these are likely to be linked. To generate a dataset of putatively neutral loci with *F*_ST_ outliers removed, we ran the filtered SNP genotype data through BayeScan 2.0 (Foll & Gaggiotti, 2008) using 20 pilot runs of 5000 iterations, followed by 100,000 iterations for sampling (Thomas et al., 2020).

### 2.2 Population genetic connectivity

The package poppr (Kamvar et al., 2014) was used to calculate genotypic diversity measures on the neutral loci dataset, and the package StAMPP (Winter, 2012) was applied to determine significance in pairwise *F*_ST_ and genetic differentiation between reef systems, reefs and sites (Nei, 1973). Furthermore, hierarchical analysis of molecular variance (AMOVA) was conducted to link variation in genetic differentiation between reef systems, reefs, sites and samples (see spatial classification in Table S1). Spatial patterns of population connectivity were estimated using discriminant analysis of principle components (DAPC) in package adegenet (Jombart, 2008). To construct the DAPC using the neutral loci dataset, optimal K was identified using the function *find*.*cluster,* retaining 600 PCs to include the highest percentage cumulative variance and lowest BIC score. Furthermore, for DAPC construction, all discriminant analysis eigenvalues were included. Additionally, the spatial structure of genotypes was investigated using fastSTRUCTURE 1.0 model‐based Bayesian clustering (Raj et al., 2014), running 100 replicates across K ranging from 1–10 (total number of reefs) on the Pawsey supercomputer facility. The ChooseK function within the fastSTRUCTURE algorithm was applied to determine the optimal K value that best explained the structure on the neutral loci dataset. The package PopGenReport (Adamack & Gruber, 2014) was used to calculate allelic richness.

### 2.3 Genetic offset to climate change

Genetic offset is a term used to describe the mismatch of gene‐environmental associations (GEAs) under future climate conditions (Bay, Harrigan, Underwood, et al., 2018; Fitzpatrick & Keller, 2015). This is usually characterized by the Euclidean distance between present and future biological space (Ellis et al., 2012). Under this framework, we used two model algorithms, gradient forest (GF) and generalized dissimilarity models (GDM) in the R packages gradientforest (Ellis et al., 2012) and gdm (Fitzpatrick et al., 2021), respectively, to describe patterns of observed genetic variation under specified climate conditions at the 26 sample sites in WA (excluding the Lalang‐garram Marine Park sites). In contrast to GF which partitions the genotype data along the gradient of environmental data, GDMs are not based on machine learning techniques and integrate distance matrices to fit gene‐environmental responses using I‐splines, which inform on the magnitude and slope of variables when explaining genetic turnover (Fitzpatrick et al., 2013; Fitzpatrick & Keller, 2015; Gibson et al., 2017). Once gene‐environmental responses were identified at sample site locations, the models were then used to estimate regional spatial similarities in genetic composition in site‐neighbouring regions to predict future mismatches in GEAs under climate change conditions (genetic offset) across reef systems in Western Australia, following the approach described in Fitzpatrick and Keller (2015).

Before running gradientforest and gdm, we identified outlier loci with significant GEAs using BayeScEnv (excluding samples from the GBR due to high genetic dissimilarity to WA samples), which is an adapted Bayesian approach that combines *F*_ST_ differentiation at loci level with the selective pressure on allele frequencies driven by environmental and geomorphological conditions (de Villemereuil & Gaggiotti, 2015; Stucki et al., 2017). Loci outside the 95% false discovery rate threshold were considered outliers possibly under directional selection, and these were included in the genetic offset analyses.

Environmental variables were selected based on their importance in delineating coral growth, settlement and survival (Table 2) (Maina et al., 2011) and can be classified into five groups; sea surface temperature (SST), SST anomalies (Zinke et al., 2018), water column optical parameters, geomorphological variables, and physical water column parameters. All variables were downscaled to the 250 m bathymetry resolution of Australia (Whiteway, 2009) using the nearest neighbour resampling approach (Gogina & Zettler, 2010) after smoothing and completing missing environmental data using kriging interpolation (Assis et al., 2018). Once downscaled, all variables were clipped to the 0–40 m bathymetry mask, representing the zone that most photic hard corals occupy (Veron & Marsh, 1988). Prior to running BayeScEnv, variables that were correlated ≥|0.80| (Mateo et al., 2013; Senaviratna & Cooray, 2019) (Pearson correlation) with other variables at site locations were excluded to avoid overfitting, whilst retaining at least one variable from each group (Tables 2 and S4). Values of the remaining less correlated variables were extracted at each site (Table S5) and standardized to absolute environmental distances, following the BayeScEnv developers' recommendation (Villemereuil & Gaggiotti, 2015). When extracted site variable data returned NA, values at the closest neighbouring pixel were used in further analyses. Transformed variables in association with allele frequencies of the SNP genotype data were integrated in BayeScEnv, applying default chain and model parameter settings (5000 iterations, 20 pilot runs and 5000 burnin length). Posterior error probability incorporating the environmental factor (PEP g) < 0.05 was applied as recommended threshold to identify potential outlier loci or putative adaptive loci.

**TABLE 2:** Environmental and geomorphological variables, considered for gene‐environment associations analyses, grouped by sea surface temperature, temperature anomalies, optical parameters, physical water column parameters and geomorphological variables

Class	Variables	Spatial resolution (km^2^)	Temporal resolution	Temporal intervals	Units	Source
**Sea surface temperature (SST)**	Mean SST mean	4.16	1982–2017	Weekly	Kelvin^a^	CorTad version 6
	Mean SST stdev	4.16	1982–2017	Weekly	Kelvin	CorTad version 6
	Mean SST min	4.16	1982–2017	Yearly	Kelvin	CorTad version 6
	**Mean SST max**	4.16	1982–2017	Yearly	Kelvin	CorTad version 6
	**Mean SST range**	4.16	1982–2017	Yearly	Kelvin	derived from Cortad version 6 data
**Temperature anomalies**	Mean thermal stress anomalies (TSA)	4.16	1982–2017	Yearly	Kelvin	CorTad version 6
	**Mean SST anomalies (SSTA)**	4.99	1985–2017	Yearly	Kelvin	Coral Reef Watch
**Optical** **parameters**	**Total suspended matter (TSM)**	4	2002–2012	Monthly	mg/m^3^	Globcolour^b^
	**Mean chlorophyll a (Chl a)** ^c^	4	2002–2012	Monthly	g/m^3^	Globcolour
	**Mean maximum light intensity at maximum depth (Light)**	9.2	2002–2014	Monthly	Einstein/m^2^/day	Bio Oracle version 2^d^
**Physical water column parameter**	**90th percentile tidal height (Tidal height)**	8.33	2008	‐	Metre	CSIRO^e^
**Geomorphological variables**	**Bathymetry (Bath)**	0.25	2009	‐	Metre	Geoscience Australia^f^
	**Terrain roughness (Rough)**	0.25	2009	‐	Degrees	Derived from bathymetry

*Note*: Variables in bold were not correlated > |0.80| and were used for seascape and genetic offset analyses.

Temperatures in Kelvin were transformed to °C for further analyses.

*GlobColour data (* http://globcolour.info *) used in this study has been developed, validated, and distributed by ACRI‐ST, France*.

Chlorophyll a in case 2 waters which represent coastal waters where inorganic particles concentration is higher than phytoplankton concentration.

Bio oracle 2 (Assis et al., 2018).

http://www.marine.csiro.au/%7Egriffin/ORE/data/ (Underwood et al., 2020).

Geoscience bathymetry layer 2009 (Whiteway, 2009).

Putatively adaptive loci were selected for the GF analysis if they were polymorphic in more than 20% of sampled populations (Fitzpatrick & Keller, 2015) while all adaptive loci, identified using BayeScEnv, were used for GDM analysis. The gradientforest algorithm was based on 2000 regression trees per SNP and constructed with a depth of conditional permutation adjusted to the number of variables (Fitzpatrick & Keller, 2015) and a variable correlation threshold of 0.8. GF model performance was calculated. Variable importance was visualized using cumulative importance plots across individual and overall SNPs with positive R^2^ values. For the GDM, the default model setting of three I‐splines was used. GDM performance was assessed based on % deviance explained and the relative variable importance was represented by the sum of I‐spline coefficients (Fitzpatrick et al., 2013).

To identify regional variation in GEA patterns and assess the future genetic offset of *A. digitifera* populations in WA, the study area of the 26 sites in WA was extended with a radius of 50 km (very few larvae disperse farther than 50 km [Graham et al., 2011; Jones et al., 2009; Underwood, 2009]). The similarity in GEAs within this 50 km radius was assessed in both models based on similarities with the environmental conditions at the sampled sites and visualized in principle component analysis (PCA) as described in Fitzpatrick and Keller (2015). As a complementary method to determine if the spatial variable importance from the gradient forest and GDM were robust, we carried out a Samβada analysis (Samβada method and results are described in the Supporting Information text in the Supporting Information data).

Finally, we used the GF and GDM models to assess the future genetic offset under climate change conditions across the different reef systems in WA. Projected SST data from four different climate change scenarios were extracted from three Atmosphere–Ocean General Circulation Models (AOGCM), CCSM4, HADGEM2‐ES and MIROC 5, from the CMIP 5 database (Taylor et al., 2012). We resampled future SST data to 4 km resolution using the NASA/OB.DAAC data analysis software (NASA SeaDAS V 7.5.3). SST data from 2040–2050 and 2090–2100 data under RCP 2.6 (mildest scenario) and RCP 8.5 (extreme case scenario) were averaged to account for variability in future SST data. Buffer zones with a radius of 50 km of future SST data were constructed using the same downscaling and masking procedures as used for the present‐day environmental conditions. Significant differences in genetic offset were tested between reef systems across the four climate change conditions using Kruskal‐wallis nonparametric tests with post hoc Bonferroni corrected Dunn test or two‐way Anova with post hoc Tukey's test based on the extracted Euclidean distance values within the 50 km radius buffer zone around the site locations.

## 3 RESULTS

### 3.1 Population genetic connectivity

**FIGURE 2:**
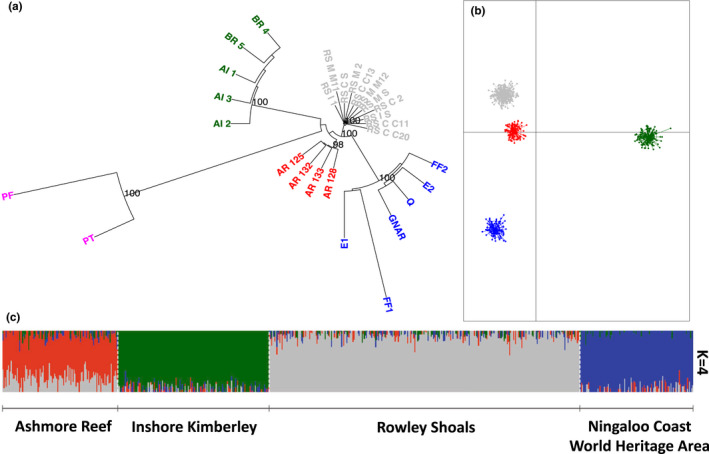
Population connectivity results. (a) Neighbour‐joining tree of all sites (labels correspond to sites which can be found in Table S1) in WA (except at Lalang‐garram Marine Park reefs) and Pelorus Island (GBR), segregating offshore NW shelf populations from Pelorus Island and inshore Kimberley populations. (b) DAPC without Pelorus Island genotypes. (c) fastSTRUCTURE admixture plot using only WA genotype data (except Lalang‐garram Marine Park genotypes) with optimal K clustering (K = 4) that best describes the population structure of the SNP data (using chooseK function). Colours correspond to reef system membership: Ashmore Reef (red), Inshore Kimberley (dark green), Rowley Shoals (grey), Ningaloo World Heritage Area (blue) and Great Barrier Reef (pink)

In total, 1550 loci across 704 samples passed quality filtering (QC) and were run through BayeScan 2.0. Overall, 1193 loci, located inside a 95% false discovery rate threshold, were considered putatively neutral loci. This neutral loci dataset was used to explore population connectivity among reef systems in Western Australia and the GBR. Across the five reef systems (including the GBR), the overall expected heterozygosity was 0.365 and the mean *F*_ST_ was 0.06 (Table 1, Table S6 and Figures S2c,d). Pairwise differences in *F*_ST_ values between reefs ranged from 0 to 0.186, and were significant in all cases except among Quobba, Gnaraloo and Ningaloo Stations sites within the Ningaloo Coast World Heritage Area, and among Imperieuse, Mermaid and Clerke reefs within the Rowley Shoals (Table S7 and S8), which are between 30–150 km apart. The highest pairwise *F*_ST_ values detected, involved Pelorus Island on GBR on the east coast and all WA reef systems (0.139–0.169) (Table S7). At the finest spatial scales (0–30 km), none of the pairwise comparisons between replicate sample sites from the same reef were significant (Table S9 and Figure S3). On the largest geographical scale, neighbour‐joining tree analyses on the neutral dataset revealed three broad groups (Figure 2a, labels correspond to the sites which can be found in Table S1); one cluster contained the offshore reefs (Ashmore Reef and Rowley Shoals) and the coastal fringing reefs within the Ningaloo Coast World Heritage Area. The second cluster comprised inshore Kimberley reefs (Adele Island and Beagle Reef), and the third cluster comprised the GBR reef system (Figure 2a). When the GBR reef system (Pelorus Island reef) was excluded, the pairwise *F*_ST_ between WA reef systems ranged between 0.02–0.081 (Table S10) (mean *F*_ST_ = 0.048; Table S11) and the reef systems in WA segregated into four distinct clusters (Figures 2b,c): Ningaloo Coast World Heritage Area, Ashmore Reef, Rowley Shoals and the inshore Kimberley, suggesting limited gene flow between these reef systems, separated by ~360 to 840 km.

### 3.2 Genetic offset to climate change

Thirteen environmental variables were initially considered in our analyses (Table 2). Nine variables remained after removing the most highly correlated (≥|0.80|) (Table 2 and Table S4). Three environmental variables were associated with temperature (mean SSTmax, mean SSTrange and sea surface temperature anomalies [SSTA]), three with water quality (total suspended matter [TSM], chlorophyll a and light intensity), and three with reef structure, substrata and oceanographic conditions (bathymetry, terrain roughness and tidal height). Thermal stress anomalies (TSA) was removed from analyses due to high collinearity with SSTrange (*r* = −0.83), which was retained because of the availability of future SSTrange data. Using BayeScEnv, we identified 110 significant gene–environmental associations across 65 unique and polymorphic loci (>20% of the sampled sites), potentially under environmental selection across our study domain, that were integrated into the GF and GDM algorithms. Of these 110 significant associations, the majority were strongly correlated to SSTmax, tidal height and SSTrange conditions across the WA reef sites (34, 21 and 18 loci, respectively).

**FIGURE 3:**
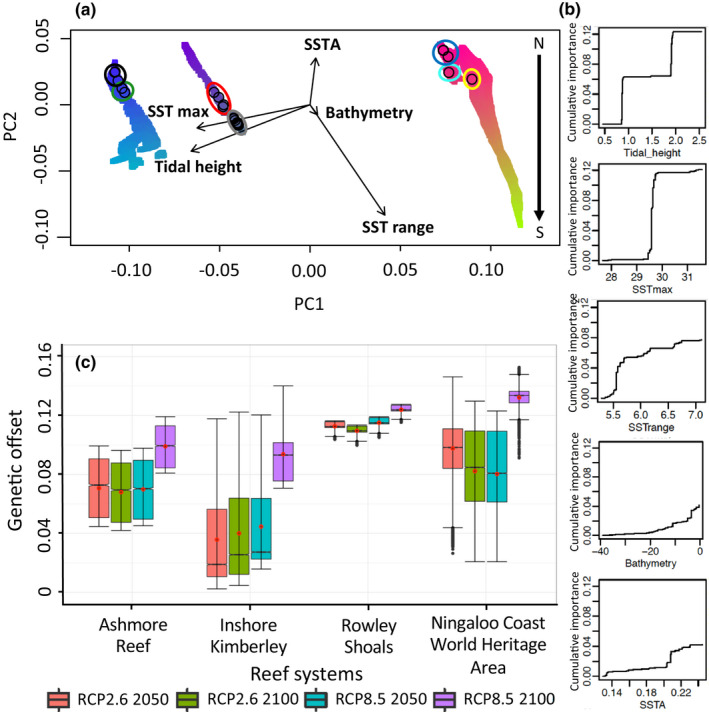
Gradient forest analysis. (a) PCA plot showing the similarity in gene‐environmental associations within the 50 km buffer zone of the sampled sites in RGB combination (red, green and blue are assigned using PC1, PC2 and PC3 combinations) using the final GF model. In this plot, the more similar the colours, the more similar areas, that neighbour sampled sites, are in terms of genetic composition with those sample sites. Vectors represent the direction and magnitude of the five most explanatory variables (SSTrange, Tidal height, SSTmax, SSTA and Bathymetry in decreasing order). Small black circles represent site locations encircled by reefs. From left to right (green – Adele Island, black – Beagle Reef (inshore Kimberley), red – Ashmore Reef, grey – Imperieuse, Clerke and Mermaid Reef (Rowley Shoals), dark blue – Ningaloo Stations, magenta – Gnaraloo, yellow – Quobba (Ningaloo Coast World Heritage Area). The N/S arrow on the right represents the latitudinal variation in genetic similarity in the Ningaloo Coast World Heritage Area as a result of the SSTrange gradient along the coastline. (b) Line plots show the trend in cumulative importance of the five most important variables to the variable distribution. (c) Notched boxplots representing the variability in the genetic offset, represented by the Euclidean distance between present‐day and future genetic composition, across reef systems in WA under RCP 2.6 and RCP 8.5 in 2040–2050 and 2090–2100 (predicted by the gradient forest model). Red circles represent mean values while black dots represent outliers

The final GF was selected after bootstrapping using minor allele frequency data from 38 of the 65 loci with positive goodness of fit (mean R^2^ = 0.457 ± 0.253). These loci were used to extrapolate the gene–environmental associations to a broader spatial scale and to examine how these associations change under future climate conditions. Variable importance for the best performing GF model was highest for tidal height, followed by SSTmax, SSTrange, bathymetry and SSTA (Figures 3, Figures S4 and S5) and these variables were used for predicting regional, spatial and temporal patterns in GEAs. In the final GDM (% deviance explained = 89.9%), tidal height and SSTmax were the only environmental variables considered significantly driving genetic variation patterns across NWA sites (Figure S6). Based on the GF and GDM analyses, we identified three distinct clusters in our dataset (Figure 3a [GF] and Figure S7 [GDM]): (1) Ningaloo Coast World Heritage Area (colours represent PCA values using RGB combinations); (2) Rowley Shoals and Ashmore Reef; (3) inshore Kimberley. This result is in contrast to the neutral dataset that clearly differentiated Rowley Shoals from Ashmore Reef populations. We found similar results in the regional pattern of environmental variable importance in the Samβada results (see Supporting Information results, Table S12), which supported GF and GDM findings. Bivariate population analysis identified K = 4 as the best population structure where 559 significant GEAs were strongly linked to SSTrange, tidal height and SSTmax across all reef systems in WA. Regionally, many GEAs were correlated with SSTrange and tidal height at Ningaloo Coast World Heritage Area reefs while a large number of significant GEAs were highly associated with tidal height, SSTrange and SSTmax in the inshore Kimberley reefs and Ashmore Reef (see Table S12).

**FIGURE 4:**
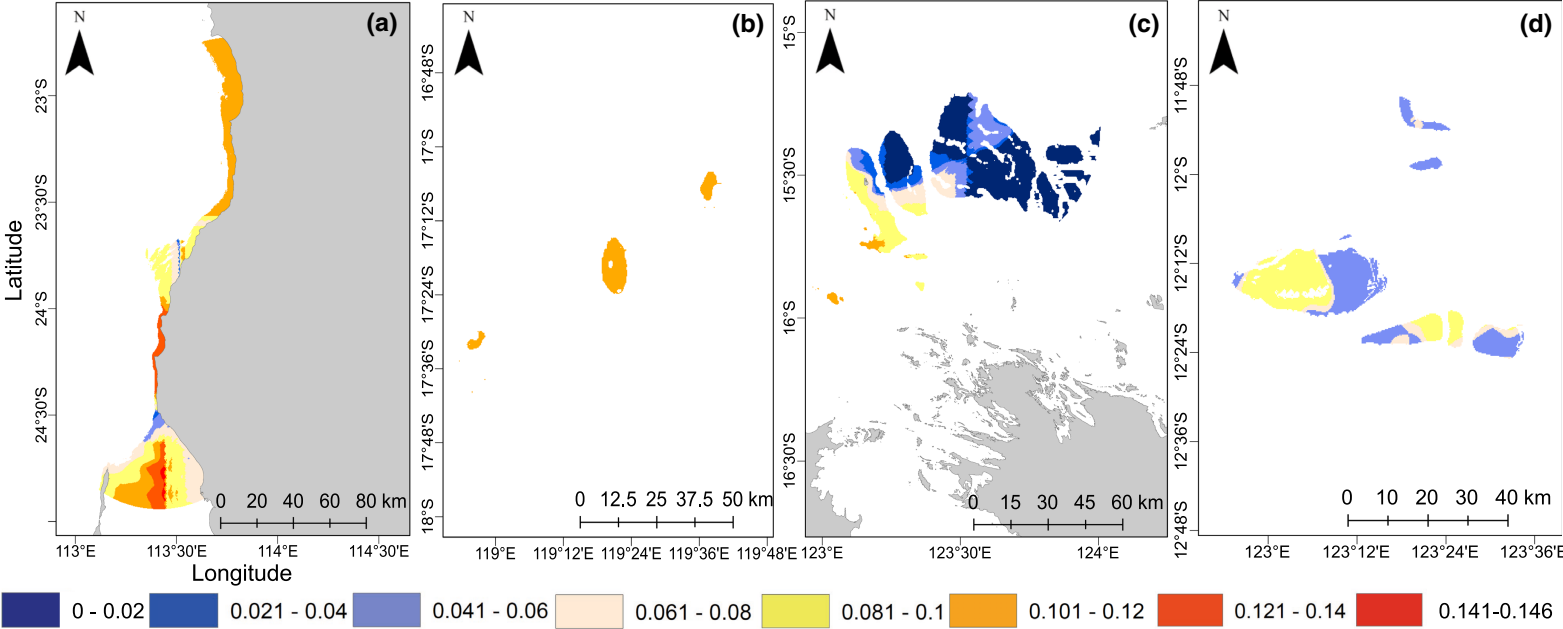
Genetic offset raster predictions, represented by the Euclidean distance between present‐day and future genetic composition, across the four reef systems in WA under RCP 2.6 climate conditions in 2040–2050, predicted using the GF model. Ningaloo Coast World Heritage Area (a), Rowley Shoals (b), inshore Kimberley (c) and Ashmore Reef (d)

All reef systems responded similarly to increasing SST across the different climate change scenarios, with the largest increase in genetic offset for all reef systems under the extreme climate change conditions (RCP 8.5 in 2090–2100) compared to RCP 8.5 in 2040–2050 and RCP 2.6 in 2040–2050 and 2090–2100 (Figure 3c, Figure S7 and Table S13). Two primary patterns emerged from our analyses. First, levels of genetic offset were significantly different among reef systems (Kruskal‐wallis *p*‐values, *p <* .001) across all climate scenarios (Figure 3c), except between Ashmore Reef and inshore Kimberley under RCP 8.5 scenario in 2040–2050 and 2090–2100 (only for GDM). For example, genetic offset under RCP 8.5 in 2090–2100 was predicted to be highest at the Ningaloo Coast World Heritage Area (0.132 ± 0.006 (GF), 0.382 ± 0.04 (GDM); mean ± deviation), and lowest in the inshore Kimberley region in the GF (0.094 ± 0.018) (Figure 3c and Table S13). The GDM predicted lowest genetic offset, across all reef systems under RCP 8.5 in 2090–2100, except in the Ningaloo Coast World Heritage Area (Table S13 and Figure S7). These patterns remained relatively consistent across the different climate scenarios, but were most pronounced in the extreme case. Secondly, we identified differences in the variance around the mean genetic offset between reef systems under the different climate change scenarios. For example, the level of variability in genetic offset in the Rowley Shoals, predicted in GF, was low compared to the other reef systems (Figures 3c and 4) while highest variability was predicted in GF and GDM in the Ningaloo Coast World Heritage Area (Figures 3c, Figure S7 and Table S13).

## 4 DISCUSSION

Using a panel of genomic‐wide SNPs to explore the genetic diversity, population structure and mismatch in future gene–environment associations of *Acropora digitifera* across 12 degrees of latitude, we identified strong population differentiation among geographically separated reef systems, indicating restricted connectivity and limited gene flow between inshore and offshore reef systems in Western Australia (WA). Loci showing strong associations with temperature revealed varying genetic offsets among different reef systems. Based on the model results presented in this study, corals living closest to their thermal stress limit in low latitude regions, such as the inshore Kimberley reef system, were predicted to require a lower adaptive shift to be able to cope with future increases in temperature, compared to mid‐latitude reefs. For example, populations in the Ningaloo Coast World Heritage Area were predicted to have pronounced gene‐environment mismatches under future climate scenarios, highlighting their vulnerability to forecasted temperature changes and the need for large and rapid adaptive shifts to keep pace with climate change. This study shows that the potential of coral populations in WA to maintain gene‐environmental associations under climate change is quite variable, complex and highly correlated with the relative regional temperature shifts, projected under climate change conditions. While the predictions of genetic offset in this study are based on future sea surface temperature conditions only, the importance of shifts in other factors such as fine scale future temperature anomalies cannot be ignored.

### 4.1 Regional genetic structure across tropical North West Australia

We identified strong regional genetic differentiation in *Acropora digitifera* among, but not within, reef systems in WA with substantial exchange of beneficial alleles within systems. Consistent with the expectations of metapopulation structure, populations from the Great Barrier Reef showed strong genetic divergence from WA samples. Within WA, distinct genetic differences were identified between populations from the offshore reef systems, the inshore macrotidal Kimberley region and Ningaloo Coast World Heritage Area reefs. The spatial patterns of restricted exchange of genetic material between reef systems are similar to that observed in other brooding and spawning coral species in northwest Australia (Rosser et al., 2020; Underwood, 2009; Underwood et al., 2018). Our data, and other studies, indicate that contemporary larval exchange between offshore reefs (Rowley Shoals and Ashmore Reef) and the inshore Kimberley reefs (Adele Island and Beagle Reef) is restricted. To sustain local populations, the reef systems examined here are reliant on self‐seeding and local recruitment to recover after disturbances and maintain population health. Hence, this study adds to the growing body of evidence highlighting the importance of local recruitment in maintaining healthy coral populations (Gilmour et al., 2013; Thomas et al., 2017; Underwood, 2009; Underwood et al., 2020, 2009). At a metapopulation scale, this dataset also highlights unexpected evolutionary linkages between the offshore NW shelf reefs and the Ningaloo Reef system, however, the strength and antiquity of those connections requires further examination.

### 4.2 Genetic offset across Western Australian reef systems

The genetic offset results indicate that the responses of Western Australian coral populations to climate change conditions are likely to be variable and spatially complex. The sensitivity and reactiveness of coral populations to changing environmental conditions have been described in the literature as fundamentally different in marine and terrestrial organisms (Burrows et al., 2011; Pinsky et al., 2019, 2013). More specifically, marine organisms have a broad and variable dispersal capacity (Kinlan & Gaines, 2003) and live close to their environmental limits. Hence, marine species are more responsive and sensitive to fluctuating environmental conditions, such as temperature anomalies, than terrestrial organisms (Pinsky et al., 2019, 2013), which in turn could affect the magnitude of future genetic offset predicted in these populations.

Our results indicate that there is variability in gene‐environmental association mismatches under a range of climate change conditions. As expected, the largest gene‐environmental mismatch was predicted under the extreme climate conditions (RCP 8.5 in 2090–2100) and revealed different degrees of genetic offset across the reef systems in WA. For example, *A. digitifera* populations at the inshore Kimberley region were predicted to experience the lowest mismatch in genetic variation under climate change conditions compared to other reef systems in WA, which supports the high resilience and adaptive potential predicted for this region in other studies (Richards et al., 2015; Underwood et al., 2020). In contrast, GEA mismatches were predicted to be highest in Ningaloo Coast World Heritage Area, indicating mismatches in local adaptive potential of these populations to increasing temperatures, especially under RCP 8.5 conditions in 2090–2100. Ningaloo has been predicted to serve as future stronghold of coral biodiversity under RCP 8.5 climate conditions in 2090–2100 (Adam et al., 2021). However, coral reefs within the Ningaloo Coast World Heritage Area have been impacted over the last decade (Gilmour et al., 2019) with parts of the reef system been damaged in recent years by mass bleaching and cyclones (Depczynski et al., 2013; Gilmour et al., 2019; Moore et al., 2012; Speed et al., 2013), with some reefs showing limited recovery (Babcock et al., 2021). Therefore, the level of GEA mismatches identified in this study may offset the potential for this region to function as future coral refugia.

Two hypotheses can be presented that could explain the pattern of genetic offset found across the study area. The first hypothesis is that the magnitude in genetic offset is strongly linked to the specific regional environmental conditions and the level of local adaptive potential to temperature conditions. More specifically, the extent that *A. digitifera* populations are adapted to their local unique environmental conditions (specifically thermal variability) is inversely related to the predicted genetic offset. This means that strong adaptation to local temperature conditions could result in lower future mismatches in gene‐environmental associations and potentially increased resilience potential. Tidal height, SSTrange and SSTmax were identified as the strongest drivers of local adaptation and could be considered key environmental variables across all reef systems in tropical WA, although their influence diminishes from low to mid latitude reef systems (see GF, GDM and Samßada results). These results reflect the variety of unique environmental conditions documented in the studied reef systems (Gilmour et al., 2019; Richards et al., 2009, 2018, 2014, 2015; Speed et al., 2013; Thomas et al., 2020; Zinke et al., 2018). For example, the inshore Kimberley is known for its specialized coral communities that are able to survive harsh and variable environmental conditions (Richards et al., 2018, 2014, 2015; Underwood et al., 2020). These coral populations are probably adapted to the high turbidity, extreme tides (>11 m) and high temperatures that are typical for the region (Richards et al., 2018, 2013; Underwood et al., 2017, 2020). In contrast, offshore reef systems such as the Rowley Shoals and Ashmore Reef are more isolated, surrounded by oligotrophic clear oceanic waters with a smaller tidal range and have experienced variable levels of heat stress over the last decade, impacting coral communities in these regions (Gilmour et al., 2019; Thomas et al., 2020; Zinke et al., 2018). Conversely, fringing reefs at the Ningaloo Coast World Heritage Area, characterized by high total suspended matter conditions, experience variable ranges of sea surface temperature conditions and frequent cyclone activity (Zinke et al., 2018).

Our results also showed that the variability in regional environmental conditions between reef systems is correlated to the spatial scale of these systems (Figure S8) as well as the spatiotemporal resolution of the variable data integrated in the models. For example, as site locations are more spread out across the Ningaloo Coast World Heritage Area and inshore Kimberley reef system, more environmental variation could be integrated into the GF and GDM models compared to smaller areas such as Ashmore Reef and the Rowley Shoals. To interpret the genetic offset results and understand the environmental processes at play, it is important to understand that the fine scale spatial and temporal microhabitat temperature type variation that we see for example at the Rowley Shoals, such as daily fluctuations in temperature, are not resolved in the GF and GDM models. Due to habitat variation (e.g., lagoon vs. outer reefs), the Rowley Shoals experience variable fine scale environmental conditions (Gilmour et al., 2019, 2022). Such fine scale spatial and temporal variation within environmental variables can have subtle yet profound impacts on the resilience potential of coral populations (Thomas et al., 2020).

The second hypothesis is that the magnitude of temperature shifts across latitude drives the regional genetic offset predictions across WA. This could explain why mid latitude reefs were predicted to experience higher genetic mismatches to cope with future climate conditions compared to those in low latitude regions. When comparing SSTmax and SSTrange conditions under present‐day and RCP 8.5 in 2090–2100 between Ningaloo Coast World Heritage Area and inshore Kimberley reef systems, we observed a dramatic shift in the magnitude of change in future temperature conditions. In particular, SSTmax within the Ningaloo Coast World Heritage Area increases from ~27–28°C to 31–32°C (Figure S8), which has also been predicted in other studies (Saha et al., 2018). In comparison, a smaller change in SSTmax was predicted (from ~31–32°C to 33.5–34°C) within the inshore Kimberley reef system (Figure S8). This shows that when coral populations are locally adapted to temperature conditions, drastic temperature shifts could result in higher predicted distances between present‐day and future genetic composition and therefore an increased genetic offset. These regional differences in future temperature conditions show that many Ningaloo reefs would need to adapt to a larger increasing temperature change than the inshore Kimberley populations. The hypothesis that inshore Kimberley coral populations are highly adapted to extreme temperature conditions which could benefit their resilience potential to future climate conditions, has been suggested previously (Richards et al., 2015). However, whether these populations have already reached their adaptive limit and therefore are restricted in their ability to persist under future temperature conditions is unknown.

Also, the GF and GDM models that were used to assess the genetic offset are associated with certain assumptions and, in some cases, these provide limits to interpretation. For example, the outcomes presented here are based on future changes across certain temperature variables (SSTmax and SSTrange), assuming no future changes in migration, reproductive success, brood stock, mutation rate and local adaptation potential, or shifts in anomalous conditions or population dynamics. All of these factors are considered to potentially influence coral reef resilience under climate change conditions but are difficult to project over time. Many studies have discussed the impact of extensive heat stress (Zinke et al., 2018, 2015), driving the large scale degradation of coral reefs and the erosion of population structure (Depczynski et al., 2013; Gilmour et al., 2019; Hughes et al., 2017; McManus et al., 2021, 2020. Underwood et al., 2007). Hence, the recovery capacity of coral populations in WA reef systems is highly dependent on the extent and frequency of anomalous heat stress events, which are predicted to intensify towards mid‐high latitude regions along the WA coastline over the next decades (van Hooidonk et al., 2016, 2014, 2013). However, future thermal stress metrics have not been integrated in the GF and GDM models to estimate future genetic offset due to high collinearity with other temperature related variables, even though thermal stress has impacted all coral reef systems investigated in this study to some extent (Gilmour et al., 2019) and is likely to have structured the local adaptive capacity of coral populations. Furthermore, an increasing body of evidence is highlighting how rising sea level (projected to increase up to 1.4 m in Fremantle, Southwest of Australia [Carson et al., 2016]) not only impacts the distribution of coral populations but also affects accretion levels of coral reefs (Cornwall et al., 2021), thereby compromising the structural integrity of these habitats. Overall, the genetic offset is sensitive to a wide array of future changes that are not easily incorporated into the models, hence re‐evaluating these with additional data is warranted.

A second potential limitation in this study, is that the selection of outlier loci was based on statistical analyses in BayeScEnv, rather than a priori knowledge of adaptive SNPs as seen in Fitzpatrick and Keller (2015). However, gathering this type of information requires a large scale controlled experimental setup (Bay, Harrigan, Buermann, et al., 2018). Hence, confounding effects of neutral loci could have influenced gene‐environmental responses in the models and have led to over‐ or underinflation of future genetic offset predictions. Other confounding factors that need to be considered include the correlation between geographic distance with differences in environmental conditions as some correlated variables appear to be identified as important variables in the GF and GDM models (Table S14 and Figure S9). This shows that more distant sites tend to be environmentally more distinct than neighbouring sites, which could potentially inflate the model predictions.

Whether the broadscale projects of gene‐environmental mismatches that we described here for *A. digitifera* are transferable to other coral species with similar or different reproductive modes is unknown. In contrast to broadcast spawning corals that release gametes in the water column that can travel over large distances, brooding corals release larvae in close proximity to the parents, which makes the latter particularly more vulnerable to changing climate conditions. Based on our findings, we hypothesise that brooding coral populations, which are highly adapted to local conditions, could experience even higher mismatches in gene‐environmental associations with the increasing rate of future temperature shifts.

Based on these projections, we can assume that coral populations at tropical reef systems in WA, which predominantly depend on local recruitment to replenish populations after disturbance events, will respond differently to climate change pressure. As the potential for populations to adapt to climate change conditions is strongly correlated with the magnitude in temporal temperature shifts, populations such as those in the inshore Kimberley showed to experience the lowest mismatch in genetic variation under future temperature shifts. While inshore Kimberley populations are predicted to experience the lowest genetic offset across reef systems in WA, it is uncertain whether these populations have the capacity to respond and adapt fast enough to keep up with increasing frequency and magnitude of temperature change. Therefore, the gene‐environmental associations analyses in this study provide the building blocks for future research to investigate rates of adaption and whether the shifts in population genetics are likely to convey greater resistance of coral reef systems to future heat stress. Nevertheless, the increased pressure of climate change, variability in environmental responses as well as spatial and genetic isolation of coral populations in WA, calls for regionally tailored conservation and management strategies to monitor how the metapopulation responds to the increased intensity of climate disturbances in the future.

## 5 CONCLUSION

This study identified an increasing vulnerability of coral populations in Western Australia to rising global temperatures. It also supports the notion that reef systems in WA are highly adapted to local environmental conditions, reproductively isolated from neighbouring systems, and therefore self‐reliant for population maintenance and genetic rescue. However, our data also revealed pronounced differences in genetic offset among our sampled reefs, offering a glimmer of hope that some reef systems, such as the inshore Kimberley, may fare better than others under climate change conditions. However, inferences about future adaptive potential for populations are based on the observed distribution of heat adapted alleles, which are strongly correlated with the background exposure to higher temperatures. Furthermore, our results show that the capacity of populations to maintain present‐day adaptive potential under climate change conditions is highly dependent on the magnitude of regional temperature change predicted in the future. Nonetheless, the primary factor determining the impact of climate change on coral reefs is the frequency and severity of temperature increases, which typically overwhelm the latent adaptive capacity of many reefs and habitats. Variation in adaptive capacity will slow the degradation of some populations on some reefs; however, reducing rates of temperature increase generated through carbon emissions remains the most effective means maintaining coral reef ecosystems into the future. Given the prediction of recurrent mass mortality events in the future, broadly evaluating the metapopulation structure and the adaptive capacity of populations provides useful information for the prioritization of limited conservation resources.

## AUTHOR CONTRIBUTIONS

Arne Adam, Luke Thomas, Jim Underwood and Zoe Richards conceived the study; Zoe Richards and James Gilmour secured project funding and arranged fieldwork logistics. James Gilmour and Luke Thomas provided data from the Rowley Shoals and Ashmore Reef. Zoe Richards and Arne Adam collected additional samples from the inshore Kimberley and Ningaloo World Heritage Area; Arne Adam conducted the data analyses. Luke Thomas, Jim Underwood, James Gilmour and Zoe Richards advised on data analyses; Arne Adam wrote the manuscript; All authors contributed to manuscript editing.

## CONFLICT OF INTEREST

All authors declare no conflict of interest.

## Supporting information


Data S1

## Data Availability

Raw sequencing data, filtered raw DArT SNP genotype data set as well as DArT filtering and genetic offset modelling scripts in R have been made available through the Dryad online data repository platform, https://doi.org/10.5061/dryad.t1g1jwt4g.

## References

[mec16498-bib-0001] Adam, A. A. S. , Garcia, R. A. , Galaiduk, R. , Tomlinson, S. , Radford, B. , Thomas, L. , & Richards, Z. T. (2021). Diminishing potential for tropical reefs to function as coral diversity strongholds under climate change conditions. Diversity and Distributions, 27(11), 2245–2261.

[mec16498-bib-0002] Adamack, A. T. , & Gruber, B. (2014). PopGenReport: Simplifying basic population genetic analyses in R. Methods in Ecology and Evolution, 5(4), 384–387.

[mec16498-bib-0003] Aranda, M. , Li, Y. , Liew, Y. J. , Baumgarten, S. , Simakov, O. , Wilson, M. C. , Piel, J. , Ashoor, H. , Bougouffa, S. , Bajic, V. B. , Ryu, T. , Ravasi, T. , Bayer, T. , Micklem, G. , Kim, H. , Bhak, J. , LaJeunesse, T. C. , & Voolstra, C. R. (2016). Genomes of coral dinoflagellate symbionts highlight evolutionary adaptations conducive to a symbiotic lifestyle. Scientific Reports, 6(1), 39734. 28004835 10.1038/srep39734 PMC5177918

[mec16498-bib-0004] Assis, J. , Tyberghein, L. , Bosch, S. , Verbruggen, H. , Serrão, E. A. , & De Clerck, O. (2018). Bio‐ORACLE v2.0: Extending marine data layers for bioclimatic modelling. Global Ecology and Biogeography, 27(3), 277–284.

[mec16498-bib-0005] Babcock, R. C. , Thomson, D. P. , Haywood, M. D. E. , Vanderklift, M. A. , Pillans, R. , Rochester, W. A. , Miller, M. , Speed, C. W. , Shedrawi, G. , & Field, S. (2021). Recurrent coral bleaching in north‐Western Australia and associated declines in coral cover. Marine and Freshwater Research, 72, 620.

[mec16498-bib-0006] Balkenhol, N. , Dudaniec, R. Y. , Krutovsky, K. V. , Johnson, J. S. , Cairns, D. M. , Segelbacher, G. , Selkoe, K. A. , von der Heyden, S. , Wang, I. J. , & Selmoni, O. (2017). Landscape genomics: understanding relationships between environmental heterogeneity and genomic characteristics of populations In Population genomics (pp. 261–322). Springer.

[mec16498-bib-0007] Bay, R. A. , Harrigan, R. J. , Buermann, W. , Underwood, V. L. , Gibbs, H. L. , Smith, T. B. , & Ruegg, K. (2018). Response to Comment on “Genomic signals of selection predict climate‐driven population declines in a migratory bird”. Science, 361(6401), eaat7956. 10.1126/science.aat7956 30072513

[mec16498-bib-0008] Bay, R. A. , Harrigan, R. J. , Underwood, V. L. , Gibbs, H. L. , Smith, T. B. , & Ruegg, K. (2018). Genomic signals of selection predict climate‐driven population declines in a migratory bird. Science, 359(6371), 83–86. 29302012 10.1126/science.aan4380

[mec16498-bib-0009] Bay, R. A. , Rose, N. H. , Logan, C. A. , & Palumbi, S. R. (2017). Genomic models predict successful coral adaptation if future ocean warming rates are reduced. Science Advances, 3(11), e1701413. 29109975 10.1126/sciadv.1701413 PMC5665595

[mec16498-bib-0010] Benkwitt, C. E. , Wilson, S. K. , & Graham, N. A. J. (2020). Biodiversity increases ecosystem functions despite multiple stressors on coral reefs. Nature Ecology and Evolution, 4(7), 919–926. 32424279 10.1038/s41559-020-1203-9

[mec16498-bib-0011] Burrows, M. T. , Schoeman, D. S. , Buckley, L. B. , Moore, P. , Poloczanska, E. S. , Brander, K. M. , Brown, C. , Bruno, J. F. , Duarte, C. M. , Halpern, B. S. , Holding, J. , Kappel, C. V. , Kiessling, W. , O'Connor, M. I. , Pandolfi, J. M. , Parmesan, C. , Schwing, F. B. , Sydeman, W. J. , & Richardson, A. J. (2011). The pace of shifting climate in marine and terrestrial ecosystems. Science, 334(6056), 652–655. 22053045 10.1126/science.1210288

[mec16498-bib-0012] Carson, M. , Köhl, A. , Stammer, D. , Slangen, A. B. A. , Katsman, C. A. , van de Wal, W. R. S. , Church, J. , & White, N. (2016). Coastal sea level changes, observed and projected during the 20th and 21st century. Climatic Change, 134(1), 269–281.

[mec16498-bib-0013] Cornwall, C. E. , Comeau, S. , Kornder, N. A. , Perry, C. T. , vanHooidonk, R. , DeCarlo, T. M. , Pratchett, M. S. , Anderson, K. D. , Browne, N. , Carpenter, R. , Diaz‐Pulido, G. , D'Olivo, J. P. , Doo, S. S. , Figueiredo, J. , Fortunato, S. A. V. , Kennedy, E. , Lantz, C. A. , McCulloch, M. T. , Gonzàlez‐Rivero, M. , … Lowe, R. J. (2021). Global declines in coral reef calcium carbonate production under ocean acidification and warming. Proceedings of the National Academy of Sciences of the United States of America, 118(21), e2015265118. 33972407 10.1073/pnas.2015265118 PMC8166140

[mec16498-bib-0014] de Villemereuil, P. , & Gaggiotti, O. E. (2015). A new FST‐based method to uncover local adaptation using environmental variables. Methods in Ecology and Evolution, 6(11), 1248–1258.

[mec16498-bib-0015] Depczynski, M. , Gilmour, J. P. , Ridgway, T. , Barnes, H. , Heyward, A. J. , Holmes, T. H. , Moore, J. A. Y. , Radford, B. T. , Thomson, D. P. , Tinkler, P. , & Wilson, S. K. (2013). Bleaching, coral mortality and subsequent survivorship on a West Australian fringing reef. Coral Reefs, 32(1), 233–238.

[mec16498-bib-0016] Dietzel, A. , Connolly, S. R. , Hughes, T. P. , & Bode, M. (2021). The spatial footprint and patchiness of large‐scale disturbances on coral reefs. Global Change Biology, 27(19), 4825–4838. 34390297 10.1111/gcb.15805

[mec16498-bib-0017] Duruz, S. , Sevane, N. , Selmoni, O. , Vajana, E. , Leempoel, K. , Stucki, S. , Orozco‐terWengel, P. , Rochat, E. , Dunner, S. , The Nextgen Consortium , The Climgen Consortium , Bruford, M. W. , & Joost, S. (2019). Rapid identification and interpretation of gene–environment associations using the new R.SamBada landscape genomics pipeline. Molecular Ecology Resources, 19(5), 1355–1365. 31136078 10.1111/1755-0998.13044 PMC6790591

[mec16498-bib-0018] Ellis, N. , Smith, S. J. , & Pitcher, C. R. (2012). Gradient forests: Calculating importance gradients on physical predictors. Ecology, 93(1), 156–168. 22486096 10.1890/11-0252.1

[mec16498-bib-0019] Fitzpatrick, M. , Mokany, K. , Manion, G. , Lisk, M. , Ferrier, S. , & Nieto‐Lugilde, D. (2021). gdm: Generalized Dissimilarity Modelling. R package version 1.4.2.2. https://CRAN.R‐project.org/package=gdm

[mec16498-bib-0020] Fitzpatrick, M. C. , & Keller, S. R. (2015). Ecological genomics meets community‐level modelling of biodiversity: Mapping the genomic landscape of current and future environmental adaptation. Ecology Letters, 18(1), 1–16. 25270536 10.1111/ele.12376

[mec16498-bib-0021] Fitzpatrick, M. C. , Sanders, N. J. , Normand, S. , Svenning, J.‐C. , Ferrier, S. , Gove, A. D. , & Dunn, R. R. (2013). Environmental and historical imprints on beta diversity: Insights from variation in rates of species turnover along gradients. Proceedings of the Royal Society B: Biological Sciences, 280(1768), 20131201. 10.1098/rspb.2013.1201 PMC3757964 23926147

[mec16498-bib-0022] Foll, M. , & Gaggiotti, O. (2008). A genome‐scan method to identify selected loci appropriate for both dominant and codominant markers: A bayesian perspective. Genetics, 180(2), 977–993. 18780740 10.1534/genetics.108.092221 PMC2567396

[mec16498-bib-0023] Gaitán‐Espitia, J. D. , & Hobday, A. J. (2021). Evolutionary principles and genetic considerations for guiding conservation interventions under climate change. Global Change Biology, 27(3), 475–488. 32979891 10.1111/gcb.15359

[mec16498-bib-0024] Gervais, C. R. , Huveneers, C. , Rummer, J. L. , & Brown, C. (2021). Population variation in the thermal response to climate change reveals differing sensitivity in a benthic shark. Global Change Biology, 27(1), 108–120. 33118308 10.1111/gcb.15422

[mec16498-bib-0025] Gibson, N. , Prober, S. , Meissner, R. , & Van Leeuwen, S. (2017). Implications of high species turnover on the south‐western Australian sandplains. PLoS One, 12(2), e0172977. 28245232 10.1371/journal.pone.0172977 PMC5330496

[mec16498-bib-0026] Gilmour, J. , Cook, K. , Ryan, N. , Puotinen, M. , Green, R. , Shedrawi, G. , Hobbs, J.‐P. , Thomson, D. , Babcock, R. , Buckee, J. , Foster, T. , Richards, Z. T. , Wilson, S. , Barnes, P. , Coutts, T. , Radford, B. , Piggott, C. , Depczynski, M. , Evans, S. , & Oades, D. (2019). The state of Western Australia's coral reefs. Coral Reefs, 38, 651–667.

[mec16498-bib-0200] Gilmour, J. P. , Cook, K. L. , Ryan, N. M. , Puotinen, M. L. , Green, R. H. , & Heyward, A. J. (2022). A tale of two reef systems: local conditions, disturbances, coral life histories, and the climate catastrophe. Ecological Applications, 32, e2509. 34870357 10.1002/eap.2509

[mec16498-bib-0027] Gilmour, J. P. , Smith, L. D. , Heyward, A. J. , Baird, A. H. , & Pratchett, M. S. (2013). Recovery of an isolated coral reef system following severe disturbance. Science, 340(6128), 69–71. 23559247 10.1126/science.1232310

[mec16498-bib-0028] Gogina, M. , & Zettler, M. L. (2010). Diversity and distribution of benthic macrofauna in the Baltic Sea: Data inventory and its use for species distribution modelling and prediction. Journal of Sea Research, 64(3), 313–321.

[mec16498-bib-0029] Graham, N. A. J. , Nash, K. L. , & Kool, J. T. (2011). Coral reef recovery dynamics in a changing world. Coral Reefs, 30(2), 283–294.

[mec16498-bib-0030] Guan, Y. , Hohn, S. , Wild, C. , & Merico, A. (2020). Vulnerability of global coral reef habitat suitability to ocean warming, acidification and eutrophication. Global Change Biology, 26(10), 5646–5660. 32713061 10.1111/gcb.15293

[mec16498-bib-0031] Hoffmann, A. A. , & Sgro, C. M. (2011). Climate change and evolutionary adaptation. Nature, 470(7335), 479–485. 21350480 10.1038/nature09670

[mec16498-bib-0032] Hughes, T. P. , Kerry, J. T. , Álvarez‐Noriega, M. , Álvarez‐Romero, J. G. , Anderson, K. D. , Baird, A. H. , Babcock, R. C. , Beger, M. , Bellwood, D. R. , Berkelmans, R. , Bridge, T. C. , Butler, I. R. , Byrne, M. , Cantin, N. E. , Comeau, S. , Connolly, S. R. , Cumming, G. S. , Dalton, S. J. , Diaz Pulido, G. , … Wilson, S. K. (2017). Global warming and recurrent mass bleaching of corals. Nature, 543, 373–377. 28300113 10.1038/nature21707

[mec16498-bib-0033] Hughes, T. P. , Kerry, J. T. , Baird, A. H. , Connolly, S. R. , Chase, T. J. , Dietzel, A. , Hill, T. , Hoey, A. S. , Hoogenboom, M. O. , Jacobson, M. , Kerswell, A. , Madin, J. S. , Mieog, A. , Paley, A. S. , Pratchett, M. S. , Troda, G. , & Woods, R. M. (2019). Global warming impairs stock–recruitment dynamics of corals. Nature, 568(7752), 387–390. 30944475 10.1038/s41586-019-1081-y

[mec16498-bib-0034] Jombart, T. (2008). adegenet: A R package for the multivariate analysis of genetic markers. Bioinformatics, 24(11), 1403–1405. 18397895 10.1093/bioinformatics/btn129

[mec16498-bib-0035] Jones, G. P. , Almany, G. R. , Russ, G. R. , Sale, P. F. , Steneck, R. S. , van Oppen, M. J. H. , & Willis, B. L. (2009). Larval retention and connectivity among populations of corals and reef fishes: History, advances and challenges. Coral Reefs, 28(2), 307–325.

[mec16498-bib-0036] Kamvar, Z. N. , Tabima, J. F. , & Grünwald, N. J. (2014). Poppr: An R package for genetic analysis of populations with clonal, partially clonal, and/or sexual reproduction. PeerJ, 2, e281. 24688859 10.7717/peerj.281 PMC3961149

[mec16498-bib-0037] Kinlan, B. P. , & Gaines, S. D. (2003). Propagule dispersal in marine and terrestrial environments: A community perspective. Ecology, 84(8), 2007–2020.

[mec16498-bib-0038] Kleypas, J. A. , Thompson, D. M. , Castruccio, F. S. , Curchitser, E. N. , Pinsky, M. , & Watson, J. R. (2016). Larval connectivity across temperature gradients and its potential effect on heat tolerance in coral populations. Global Change Biology, 22(11), 3539–3549. 27154763 10.1111/gcb.13347

[mec16498-bib-0039] Lin, S. , Cheng, S. , Song, B. , Zhong, X. , Lin, X. , Li, W. , Li, L. , Zhang, Y. , Zhang, H. , Ji, Z. , Cai, M. , Zhuang, Y. , Shi, X. , Lin, L. , Wang, L. , Wang, Z. , Liu, X. , Yu, S. , Zeng, P. , & Morse, D. (2015). The *Symbiodinium kawagutii* genome illuminates dinoflagellate gene expression and coral symbiosis. Science, 350(6261), 691–694. 26542574 10.1126/science.aad0408

[mec16498-bib-0040] Liu, H. , Stephens, T. G. , González‐Pech, R. A. , Beltran, V. H. , Lapeyre, B. , Bongaerts, P. , Cooke, I. , Aranda, M. , Bourne, D. G. , Forêt, S. , Miller, D. J. , & Chan, C. X. (2018). Symbiodinium genomes reveal adaptive evolution of functions related to coral‐dinoflagellate symbiosis. Communications Biology, 1(1), 95. 30271976 10.1038/s42003-018-0098-3 PMC6123633

[mec16498-bib-0041] Maina, J. , McClanahan, T. R. , Venus, V. , Ateweberhan, M. , & Madin, J. (2011). Global gradients of coral exposure to environmental stresses and implications for local management. PLoS One, 6(8), e23064. 21860667 10.1371/journal.pone.0023064 PMC3156087

[mec16498-bib-0042] Malhi, Y. , Franklin, J. , Seddon, N. , Solan, M. , Turner, M. G. , Field, C. B. , & Knowlton, N. (2020). Climate change and ecosystems: Threats, opportunities and solutions. Philosophical Transactions of the Royal Society B: Biological Sciences, 375(1794), 20190104. 10.1098/rstb.2019.0104 PMC7017779 31983329

[mec16498-bib-0043] Mateo, R. G. , Vanderpoorten, A. , Muñoz, J. , Laenen, B. , & Désamoré, A. (2013). Modeling species distributions from heterogeneous data for the biogeographic regionalization of the European bryophyte flora. PLoS One, 8(2), e55648. 23409015 10.1371/journal.pone.0055648 PMC3569459

[mec16498-bib-0044] Matz, M. V. , Treml, E. A. , & Haller, B. C. (2020). Estimating the potential for coral adaptation to global warming across the Indo‐West Pacific. Global Change Biology, 26, 3473–3481. 32285562 10.1111/gcb.15060

[mec16498-bib-0045] McManus, L. C. , Forrest, D. L. , Tekwa, E. W. , Schindler, D. E. , Colton, M. A. , Webster, M. M. , Essington, T. E. , Palumbi, S. R. , Mumby, P. J. , & Pinsky, M. L. (2021). Evolution and connectivity influence the persistence and recovery of coral reefs under climate change in the Caribbean, Southwest Pacific, and Coral Triangle. Global Change Biology, 27(18), 4307–4321. 34106494 10.1111/gcb.15725 PMC8453988

[mec16498-bib-0046] McManus, L. C. , Vasconcelos, V. V. , Levin, S. A. , Thompson, D. M. , Kleypas, J. A. , Castruccio, F. S. , Curchitser, E. N., & Watson, J. R. (2020). Extreme temperature events will drive coral decline in the Coral Triangle. Global Change Biology, 26(4), 2120–2133. 10.1111/gcb.14972 31883173

[mec16498-bib-0047] Moore, J. A. Y. , Bellchambers, L. M. , Depczynski, M. R. , Evans, R. D. , Evans, S. N. , Field, S. N. , Friedman, K. J. , Gilmour, J. P. , Holmes, T. H. , Middlebrook, R. , Radford, B. T. , Ridgway, T. , Shedrawi, G. , Taylor, H. , Thomson, D. P. , & Wilson, S. K. (2012). Unprecedented mass bleaching and loss of coral across 12° of Latitude in Western Australia in 2010–11. PLoS One, 7(12), e51807. 23284773 10.1371/journal.pone.0051807 PMC3524109

[mec16498-bib-0048] Nei, M. (1973). Analysis of gene diversity in subdivided populations. Proceedings of the National Academy of Sciences of the United States of America, 70(12), 3321–3323. 4519626 10.1073/pnas.70.12.3321 PMC427228

[mec16498-bib-0049] Oscar, E. G. (2017). Metapopulations of marine species with larval dispersal: A counterpoint to Ilkka's glanville fritillary metapopulations. Annales Zoologici Fennici, 54(1–4), 97–112.

[mec16498-bib-0050] Osman, E. O. , Smith, D. J. , Ziegler, M. , Kürten, B. , Conrad, C. , El‐Haddad, K. M. , Voolstra, C. R. , & Suggett, D. J. (2018). Thermal refugia against coral bleaching throughout the northern Red Sea. Global Change Biology, 24(2), e474–e484. 29044761 10.1111/gcb.13895

[mec16498-bib-0051] Pauls, S. U. , Nowak, C. , Bálint, M. , & Pfenninger, M. (2013). The impact of global climate change on genetic diversity within populations and species. Molecular Ecology, 22(4), 925–946. 23279006 10.1111/mec.12152

[mec16498-bib-0052] Pinsky, M. L. , Eikeset, A. M. , McCauley, D. J. , Payne, J. L. , & Sunday, J. M. (2019). Greater vulnerability to warming of marine versus terrestrial ectotherms. Nature, 569(7754), 108–111. 31019302 10.1038/s41586-019-1132-4

[mec16498-bib-0053] Pinsky, M. L. , Worm, B. , Fogarty, M. J. , Sarmiento, J. L. , & Levin, S. A. (2013). Marine taxa track local climate velocities. Science, 341(6151), 1239–1242. 24031017 10.1126/science.1239352

[mec16498-bib-0054] Raj, A. , Stephens, M. , & Pritchard, J. K. (2014). fastSTRUCTURE: Variational inference of population structure in large SNP data sets. Genetics, 197(2), 573–589. 24700103 10.1534/genetics.114.164350 PMC4063916

[mec16498-bib-0055] Rellstab, C. , Gugerli, F. , Eckert, A. J. , Hancock, A. M. , & Holderegger, R. (2015). A practical guide to environmental association analysis in landscape genomics. Molecular Ecology, 24(17), 4348–4370. 26184487 10.1111/mec.13322

[mec16498-bib-0056] Richards, Z. , Beger, M. , Hobbs, J.‐P. , Bowling, T. , Chong‐Seng, K. , & Pratchett, M. (2009). Ashmore Reef National Nature Reserve and Cartier Island Marine Reserve Marine Survey 2009. In ARC Centre of Excellence for Coral Reef Studies. Produced for the Department of the Environment, Water, Heritage and the Arts. James Cook University.

[mec16498-bib-0057] Richards, Z. , Bryce, M. , & Bryce, C. (2018). The composition and structure of shallow benthic reef communities in the Kimberley, north‐west Australia. Records of the Western Australian Museum Supplement, 85, 103.

[mec16498-bib-0058] Richards, Z. , Sampey, A. , & Marsh, L. (2014). Kimberley marine biota. Historical data: Scleractinian corals. Records of the Western Australian Museum Supplement, 84(11), 111–132.

[mec16498-bib-0059] Richards, Z. T. , Bryce, M. , & Bryce, C. (2013). New records of atypical coral reef habitat in the Kimberley, Australia. Journal of Marine Biology, 2013, 1–8.

[mec16498-bib-0060] Richards, Z. T. , Garcia, R. A. , Wallace, C. C. , Rosser, N. L. , & Muir, P. R. (2015). A diverse assemblage of reef corals thriving in a dynamic intertidal reef setting (Bonaparte Archipelago, Kimberley, Australia). PLoS One, 10(2), e0117791. 25714443 10.1371/journal.pone.0117791 PMC4340616

[mec16498-bib-0061] Richards, Z. T. , Juszkiewicz, D. J. , & Hoggett, A. (2021). Spatio‐temporal persistence of scleractinian coral species at Lizard Island, Great Barrier Reef. Coral Reefs, 40, 1369–1378.

[mec16498-bib-0062] Riginos, C. , Crandall, E. D. , Liggins, L. , Bongaerts, P. , & Treml, E. A. (2016). Navigating the currents of seascape genomics: How spatial analyses can augment population genomic studies. Current Zoology, 62(6), 581–601. 29491947 10.1093/cz/zow067 PMC5804261

[mec16498-bib-0063] Riginos, C. , & Liggins, L. (2013). Seascape genetics: Populations, individuals, and genes marooned and adrift. Geography Compass, 7(3), 197–216.

[mec16498-bib-0064] Rosser, N. L. , Edyvane, K. , Malina, A. C. , Underwood, J. N. , & Johnson, M. S. (2020). Geography and spawning season drive genetic divergence among populations of the hard coral *Acropora tenuis* from Indonesia and Western Australia. Coral Reefs, 39, 989–999.

[mec16498-bib-0065] Saha, K. , Zhao, X. , Zhang, H.‐M. , Casey, K. S. , Zhang, D. , Zhang, Y. , Baker‐Yeboah, S. , Relph, J. M. , Krishnan, A. , & Ryan, T. (2018). The Coral Reef Temperature Anomaly Database (CoRTAD) Version 6 ‐ Global, 4 km Sea Surface Temperature and Related Thermal Stress Metrics for 1982 to 2018. SST, TSA. NOAA National Centers for Environmental Information.

[mec16498-bib-0066] Selkoe, K. A. , Gaggiotti, O. E. , Treml, E. A. , Wren, J. L. , Donovan, M. K. , Hawai'i Reef Connectivity C onsortium & Toonen, R. J. (2016). The DNA of coral reef biodiversity: predicting and protecting genetic diversity of reef assemblages. Proceedings of the Biological Sciences, 283(1829), 20160354. 10.1098/rspb.2016.0354 PMC4855387 27122569

[mec16498-bib-0067] Selmoni, O. , Rochat, E. , Lecellier, G. , Berteaux‐Lecellier, V. , & Joost, S. (2020). Seascape genomics as a new tool to empower coral reef conservation strategies: An example on north‐western Pacific *Acropora digitifera* . Evolutionary Applications, 13(8), 1923–1938. 32908595 10.1111/eva.12944 PMC7463334

[mec16498-bib-0068] Senaviratna, N. A. M. R. , & Cooray, T. M. J. A. (2019). Diagnosing multicollinearity of logistic regression model. Asian Journal of Probability and Statistics, 5(2), 1–9.

[mec16498-bib-0069] Shinzato, C. , Shoguchi, E. , Kawashima, T. , Hamada, M. , Hisata, K. , Tanaka, M. , Fujie, M. , Fujiwara, M. , Koyanagi, R. , Ikuta, T. , Fujiyama, A. , Miller, D. J. , & Satoh, N. (2011). Using the *Acropora digitifera* genome to understand coral responses to environmental change. Nature, 476, 320–323. 21785439 10.1038/nature10249

[mec16498-bib-0070] Shoguchi, E. , Beedessee, G. , Tada, I. , Hisata, K. , Kawashima, T. , Takeuchi, T. , Arakaki, N. , Fujie, M. , Koyanagi, R. , Roy, M. C. , Kawachi, M. , Hidaka, M. , Satoh, N. , & Shinzato, C. (2018). Two divergent Symbiodinium genomes reveal conservation of a gene cluster for sunscreen biosynthesis and recently lost genes. BMC Genomics, 19(1), 458. 29898658 10.1186/s12864-018-4857-9 PMC6001144

[mec16498-bib-0071] Shoguchi, E. , Shinzato, C. , Kawashima, T. , Gyoja, F. , Mungpakdee, S. , Koyanagi, R. , Takeuchi, T. , Hisata, K. , Tanaka, M. , Fujiwara, M. , Hamada, M. , Seidi, A. , Fujie, M. , Usami, T. , Goto, H. , Yamasaki, S. , Arakaki, N. , Suzuki, Y. , Sugano, S. , & Satoh, N. (2013). Draft assembly of the *Symbiodinium minutum* nuclear genome reveals dinoflagellate gene structure. Current Biology, 23(15), 1399–1408. 23850284 10.1016/j.cub.2013.05.062

[mec16498-bib-0072] Speed, C. W. , Babcock, R. C. , Bancroft, K. P. , Beckley, L. E. , Bellchambers, L. M. , Depczynski, M. , Field, S. N. , Friedman, K. J. , Gilmour, J. P. , Hobbs, J.‐P. A. , Kobryn, H. T. , Moore, J. A. Y. , Nutt, C. D. , Shedrawi, G. , Thomson, D. P. , & Wilson, S. K. (2013). Dynamic stability of coral reefs on the West Australian Coast. PLoS One, 8(7), e69863. 23922829 10.1371/journal.pone.0069863 PMC3726730

[mec16498-bib-0073] Stucki, S. , Orozco‐terWengel, P. , Forester, B. R. , Duruz, S. , Colli, L. , Masembe, C. , Negrini, R. , Landguth, E. , Jones, M. R. , The Nextgen Consortium , Bruford, M. W. , Taberlet, P. , & Joost, S. (2017). High performance computation of landscape genomic models including local indicators of spatial association. Molecular Ecology Resources, 17(5), 1072–1089. 27801969 10.1111/1755-0998.12629

[mec16498-bib-0074] Suggett, D. J. , & Smith, D. J. (2020). Coral bleaching patterns are the outcome of complex biological and environmental networking. Global Change Biology, 26(1), 68–79. 31618499 10.1111/gcb.14871

[mec16498-bib-0075] Taylor, K. E. , Stouffer, R. J. , & Meehl, G. A. (2012). An overview of CMIP5 and the experiment design. Bulletin of the American Meteorological Society, 93(4), 485–498.

[mec16498-bib-0076] Thomas, L. , Kennington, W. J. , Evans, R. D. , Kendrick, G. A. , & Stat, M. (2017). Restricted gene flow and local adaptation highlight the vulnerability of high‐latitude reefs to rapid environmental change. Global Change Biology, 23(6), 2197–2205. 28132420 10.1111/gcb.13639

[mec16498-bib-0077] Thomas, L. , Kennington, W. J. , Stat, M. , Wilkinson, S. P. , Kool, J. T. , & Kendrick, G. A. (2015). Isolation by resistance across a complex coral reef seascape. Proceedings of the Biological Sciences, 282(1812), 20151217. 10.1098/rspb.2015.1217 PMC4528533 26224707

[mec16498-bib-0078] Thomas, L. , Underwood, J. N. , Adam, A. A. S. , Richards, Z. T. , Dugal, L. , Miller, K. J. , & Gilmour, J. P. (2020). Contrasting patterns of genetic connectivity in brooding and spawning corals across a remote atoll system in northwest Australia. Coral Reefs, 39(1), 55–60.

[mec16498-bib-0079] Treml, E. A. , Roberts, J. J. , Chao, Y. , Halpin, P. N. , Possingham, H. P. , & Riginos, C. (2012). Reproductive output and duration of the pelagic larval stage determine seascape‐wide connectivity of marine populations. Integrative and Comparative Biology, 52(4), 525–537. 22821585 10.1093/icb/ics101

[mec16498-bib-0080] Underwood, J. , Richards, Z. , Berry, O. , & Gilmour, J. (2017). Population connectivity and genetic diversity in brooding and broadcast spawning corals in the Kimberleys. In Report of Project 1.1.3 ‐ Project 1.1.3.1 prepared for the Kimberley Marine Research Program (p. 48). Western Australian Marine Science Institution.

[mec16498-bib-0081] Underwood, J. N. (2009). Genetic diversity and divergence among coastal and offshore reefs in a hard coral depend on geographic discontinuity and oceanic currents. Evolutionary Applications, 2(2), 222–233. 25567863 10.1111/j.1752-4571.2008.00065.x PMC3352373

[mec16498-bib-0082] Underwood, J. N. , Richards, Z. , Berry, O. , Oades, D. , Howard, A. , & Gilmour, J. P. (2020). Extreme seascape drives local recruitment and genetic divergence in brooding and spawning corals in remote northwest Australia. Evolutionary Applications, 13, 2404–2421. 33005230 10.1111/eva.13033 PMC7513722

[mec16498-bib-0083] Underwood, J. N. , Richards, Z. T. , Miller, K. J. , Puotinen, M. L. , & Gilmour, J. P. (2018). Genetic signatures through space, time and multiple disturbances in a ubiquitous brooding coral. Molecular Ecology, 27(7), 1586–1602. 29575282 10.1111/mec.14559

[mec16498-bib-0084] Underwood, J. N. , Smith, L. D. , van Oppen, M. J. H. , & Gilmour, J. P. (2007). Multiple scales of genetic connectivity in a brooding coral on isolated reefs following catastrophic bleaching. Molecular Ecology, 16(4), 771–784. 17284210 10.1111/j.1365-294X.2006.03187.x

[mec16498-bib-0085] Underwood, J. N. , Smith, L. D. , van Oppen, M. J. H. , & Gilmour, J. P. (2009). Ecologically relevant dispersal of corals on isolated reefs: implications for managing resilience. Ecological Applications, 19(1), 18–29. 19323171 10.1890/07-1461.1

[mec16498-bib-0086] Underwood, J. N. , Souter, P. B. , Ballment, E. R. , Lutz, A. H. , & van Oppen, M. J. H. (2006). Development of 10 polymorphic microsatellite markers from herbicide‐bleached tissues of the brooding pocilloporid coral *Seriatopora hystrix* . Molecular Ecology Notes, 6(1), 176–178.

[mec16498-bib-0087] Underwood, J. N. , Wilson, S. K. , Ludgerus, L. , & Evans, R. D. (2013). Integrating connectivity science and spatial conservation management of coral reefs in north‐west Australia. Journal for Nature Conservation, 21(3), 163–172.

[mec16498-bib-0088] van Hooidonk, R. , Maynard, J. , Tamelander, J. , Gove, J. , Ahmadia, G. , Raymundo, L. , Williams, G. , Heron, S. F. , & Planes, S. (2016). Local‐scale projections of coral reef futures and implications of the Paris Agreement. Scientific Reports, 6(1), 39666. 28000782 10.1038/srep39666 PMC5175274

[mec16498-bib-0089] van Hooidonk, R. , Maynard, J. A. , Manzello, D. , & Planes, S. (2014). Opposite latitudinal gradients in projected ocean acidification and bleaching impacts on coral reefs. Global Change Biology, 20(1), 103–112. 24151155 10.1111/gcb.12394

[mec16498-bib-0090] van Hooidonk, R. , Maynard, J. A. , & Planes, S. (2013). Temporary refugia for coral reefs in a warming world. Nature Climate Change, 3(5), 508–511.

[mec16498-bib-0091] Veron, J. , Devantier, L. M. , Turak, E. , Green, A. L. , Kininmonth, S. , Stafford‐Smith, M. , & Peterson, N. (2009). Delineating the coral triangle. Galaxea, Journal of Coral Reef Studies, 11(2), 91–100.

[mec16498-bib-0092] Veron, J. E. N. , & Marsh, L. M. (1988). Hermatypic corals of Western Australia: records and annotated species list (Vol. 29). Western Australian Museum. Records of the Western Australian Museum Supplement No. 29.

[mec16498-bib-0093] Wallace, C. C. (1999). Staghorn corals of the world: a revision of the coral genus Acropora (Scleractinia; Astrocoeniina; Acroporidae) worldwide, with emphasis on morphology, phylogeny and biogeography. CSIRO publishing.

[mec16498-bib-0094] Whiteway, T. (2009). Australian bathymetry and topography grid. G. Australia. Retrieved from: https://data.gov.au/data/dataset/australian‐bathymetry‐and‐topography‐grid‐june‐2009.

[mec16498-bib-0095] Winter, D. J. (2012). mmod: an R library for the calculation of population differentiation statistics. Molecular Ecology Resources, 12(6), 1158–1160. 22883857 10.1111/j.1755-0998.2012.03174.x

[mec16498-bib-0096] Wood, G. , Marzinelli, E. M. , Campbell, A. H. , Steinberg, P. D. , Vergés, A. , & Coleman, M. A. (2021). Genomic vulnerability of a dominant seaweed points to future‐proofing pathways for Australia's underwater forests. Global Change Biology, 27(10), 2200–2212. 33511779 10.1111/gcb.15534

[mec16498-bib-0097] Zinke, J. , Gilmour, J. P. , Fisher, R. , Puotinen, M. , Maina, J. , Darling, E. , Stat, M. , Richards, Z. T. , McClanahan, T. R. , Beger, M. , Moore, C. , Graham, N. A. J. , Feng, M. , Hobbs, J.‐P. A. , Evans, S. N. , Field, S. , Shedrawi, G. , Babcock, R. C. , & Wilson, S. K. (2018). Gradients of disturbance and environmental conditions shape coral community structure for south‐eastern Indian Ocean reefs. Diversity and Distributions, 24(5), 605–620.

[mec16498-bib-0098] Zinke, J. , Hoell, A. , Lough, J. M. , Feng, M. , Kuret, A. J. , Clarke, H. , Ricca, V. , Rankenburg, K. , & McCulloch, M. T. (2015). Coral record of southeast Indian Ocean marine heatwaves with intensified Western Pacific temperature gradient. Nature Communications, 6, 8562. 10.1038/ncomms9562 PMC4639796 26493738

