# Supplementary material for: Population connectivity and genetic offset in the spawning coral Acropora digitifera in Western Australia
Source: Mol Ecol. 2022 Jun 5;31(13):3533–47. doi: 10.1111/mec.16498 (PMC9328316; doi:10.1111/mec.16498)
Supplement: Supplementary file 1 — Data S1 [file MEC-31-3533-s001.docx]

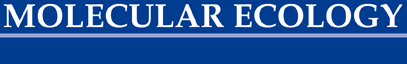


**Supplementary data for:**

**Population connectivity and genetic offset in the spawning coral *Acropora digitifera* in Western Australia**

Arne A.S. Adam ^1,2^, Luke Thomas ^2,3^, Jim Underwood ^2^, James Gilmour ^2^, Zoe T. Richards ^1,4^

^1^ Coral Conservation and Research Group, Trace and Environmental DNA Laboratory, School of Molecular and Life Sciences, Curtin University, Bentley 6102, Western Australia

^2^ Australian Institute of Marine Science, IOMRC, The University of Western Australia, Crawley 6009, Western Australia

^3^ The UWA Oceans Institute, Oceans Graduate School, The University of Western Australia, Crawley 6009, Western Australia

^4^ Collections and Research, Western Australian Museum, Welshpool, 6106, Western Australia

Corresponding Author: Arne A. S. Adam

Email: [arne.adam@postgrad.curtin.edu.au](mailto:arne.adam@postgrad.curtin.edu.au)

**Supplementary text**

**Samβada methods**

Samβada is software that investigates patterns of adaptive variation within large genomic datasets and associates these outlier loci signatures with spatial patterns in population structure and gene-environmental associations (Stucki et al., 2017). Samβada is able to quickly process large genomic datasets where each loci is modelled independently to estimate the probability of the presence of each genotype to be associated with each environmental condition, at each site (Stucki et al., 2017). For Samβada, we used the pre-processing, run and postprocessing functions in package ‘R.Samβada’ (Duruz et al., 2019). Samβada requires pre-processing of the environmental variables and genotype data in a format that can be read into the software. For the genetic data, the DArT SNP genotype genlight object (in GDS format) after QC (same input as in BayeScan and BayScEnv) was converted into a genomic matrix using the ‘prepareGeno’ function using the same QC filtering parameters as described in the methods (MAF < 0.05, missing data rate > 0.3). Only one SNP was removed after QC filtering of genomic file with 1560 SNPs remaining, resulting in 4,680 genotypes to be evaluated in Samβada across 11 variables (nine environmental/geomorphological variables + latitude and longitude). For the environmental data, final environmental file was constructed using the ‘prepareEnv’ function. Thereby, extracted environmental/geomorphological values, associated with each sample site, were formatted using the genotype ID in the genomic matrix with QC filtering (MAF < 0.05, missing data rate > 0.3, maximum correlation of variables > 0.8 and linkage disequilibrium threshold of 0.2). This function also allows for bivariate analyse for population structure analysis. The population structure is calculated based on the optimal number of PCs in the PCA that explains the highest proportion of explained variance and is used to assign a population membership coefficient for each sample in the final environmental Samβada file (Duruz et al., 2019). After analysing the population structure, three PCs were selected as the optimal number of PCs to explain the population structure and assign population membership coefficients (Figure S10). In total, 154,440 associations were evaluated using bivariate analyses into Samβada (4,680 genotypes x 11 variables x three PC population membership coefficients). For every association model, p-values of 0.05 were corrected using the Bonferroni correction method for multiple testing. Furthermore, only associations that had qG-scores and Wald scores < 0.001 were selected as significant (Selmoni et al., 2020). When multiple GEAs were identified for one genotype, only one association was selected based on the highest G-values (Stucki et al., 2017).

**Samβada results**

During pre-processing of the final environmental data for Samβada with bivariate population structure analysis, we identified PC = 3 (Figure S10) as the optimal number to show the highest proportion of variance in the population structure, clustering four populations (K = 4); 1) Ningaloo Coast World Heritage Area, 2) Rowley Shoals, 3) inshore Kimberley and 4) Ashmore Reef system. Variables, strongly correlated with the population structure were tidal height, SSTmax and SSTA, which were strongly correlated with pop 2 (R^2^ = 70.7%, 94.8% and -70.7%, respectively). After post-processing of the Samβada output, 7,046 GEAs out of 154,440 were found significant (*P <* 0.05 after Bonferroni correction, qG and qW < 0.001). These associations were calculated over 11 variables across 642 unique loci that can be identified as putative adaptive loci in Samβada. After selecting the associations with the highest G score for each variable within each population membership group (pop 1, pop 2 and pop 3) (Stucki et al., 2017), 463 GEAs were linked to population pop 1, 401 to population pop 2 and 230 to population pop 3. Across the GEAs, SSTrange, tidal height and SSTmax showed to be the most important environmental drivers of outlier genotype structure across WA sites (232, 226 and 101 GEAs, respectively, Table S12). Classifying the four reef systems in WA according to the highest population membership coefficient (Ningaloo Coast World Heritage Area – pop 1 and pop 3, Rowley Shoals- pop 1, pop 2, Inshore Kimberley -pop 1, pop 2, and pop 3 and Ashmore Reef- pop 2 and pop 3) across all population memberships and 1,094 significant GEA of putative adaptive genotypes selected in Samβada, the majority of GEAs were linked to tidal height and SST range in the Ningaloo Coast World Heritage Area reef system while for the offshore reefs and inshore Kimberley region (especially, Adele Island, Beagle Reef and Ashmore Reef system), tidal height and SST range could be identified as most important drivers of significant GEAs (Table S12).

**Table of Content**

**Supplementary Tables**

| **Table S1.** Generic and genetic metrics of all sample sites in WA (except the five Lalang-garram Marine Park sites) and Pelorus Island (GBR) | Page 5 |
| --- | --- |
| **Table S2.** Pairwise *F*_ST_ values between all five reef systems (including Lalang-garram Marine Park samples in the inshore Kimberley region) | Page 6 |
| **Table S3.** Pairwise *F*_ST_ values between all 14 reefs (including Lalang-  garram Marine Park samples in the inshore Kimberley region) | Page 7 |
| **Table S4.** Correlation of environmental and geomorphological variable data, that were considered for BayeScEnv, Samßada and genetic offset models (GF and GDM), using data from WA sample site locations (based on Pearson correlation threshold of 80%). | Page 8 |
| **Table S5.** Site coordinates (Lat-latitude, Lon-longitude) along with extracted environmental and geomorphological values (variable abbreviations correspond to the variables in Table 1) of less correlated variables that have been used in BayeScEnv, Samßada and genetic offset model analyses | Page 9-10 |
| **Table S6.** Analysis of Molecular Variance (AMOVA) differentiation among reef systems, reefs, sites and samples (without Lalang-garram Marine Park samples) | Page 10 |
| **Table S7.** Pairwise *F*_ST_ values between five reef systems (without Lalang-garram Marine Park samples) | Page 10 |
| **Table S8.** Pairwise *F*_ST_ values between all reefs (without Lalang-garram Marine Park samples) | Page 11 |
| **Table S9.** Pairwise *F*_ST_ values between all 28 sites (without Lalang-Garram Marine Park samples) | Page 12 |
| **Table S10.** Pairwise *F*_ST_ values between four reef systems (without Lalang- garram Marine Park and Great Barrier Reef samples) | Page 13 |
| **Table S11.** Analysis of Molecular Variance (AMOVA) differentiation among reef systems, reefs, sites and samples in WA (without Lalang-garram Marine Park and Great Barrier Reef samples) | Page 13 |
| **Table S12.** Samβada output of significant gene-environment associations (GEAs) after Bonferroni correction and highest G score filtering for each genotype-environmental combination | Page 13 |
| **Table S13.** Genetic offset predictions among reef systems in WA for each future climate scenarios using gradient forest (GF) and generalised dissimilarity model (GDM). | Page 14-15 |
| **Table S14.** Mantel correlation test values, evaluating the correlation between geographic and environmental distance matrices. | Page 16 |

**Supplementary Figures**

| **Figure S1**. DAPC clustering of all 14 reefs in WA and Great Barrier Reef (Pelorus Island) using SNP genotype data filtered with loci call rate (> 0.8) and individual call rate (> 0.3) parameters | Page 17 |
| --- | --- |
| **Figure S2**. Quality score filtering plots of DArT SNP genotype data | Page 18 |
| **Figure S3**. Fine spatial scale DAPC plots | Page 19 |
| **Figure S4**. Variable importance of GF model algorithm, constructed by integrating minor allele frequencies of 38 outlier loci with extracted environmental and habitat variables at every site | Page 20 |
| **Figure S5.** Cumulative variable importance of environmental and geomorphological variables in the final GF model, explaining the genetic signature across the sampled sites in WA for each of the 38 individual outlier loci with positive weighted R^2^ goodness of fit | Page 21 |
| **Figure S6.** Relative variable importance of GDM, fitted by I-splines, with tidal height, SSTmax, geographic distance as only significant contributing variables | Page 21 |
| **Figure S7.** PCA showing the similarity in gene-environmental associations within the 50 km buffer zone of the 26 sampled sites (red, green and blue are assigned using PC1, PC2 and PC3 combinations) and variability in the genetic offset predictions across reef systems in WA, using the best GDM | Page 22 |
| **Figure S8.** Regional variability in present-day and future (RCP 8.5 in 2090-2100) SSTmax and SSTrange data across the reef systems in WA | Page 22 |
| **Figure S9.** Regression plots showing top three highest significant correlations between geographic distance across sites and difference in environmental data. | Page 23 |
| **Figure S10.** Samβada population clustering plots | Page 24 |

**Supplementary Tables**

**Table S1.** Generic and genetic metrics of all sample sites in WA (except the five Lalang-garram Marine Park sites) and Pelorus Island (GBR). Total number of samples after QC (N), Expected Heterozygosity (*H*_T_), mean allelic richness (*A*_R_) at each site.

| **Reef system** | **Reefs** | **Sites** | **N** | ***H*_T_** | ***A*_R_** |
| --- | --- | --- | --- | --- | --- |
| **Ashmore Reef** | **Ashmore Reef** | Ashmore_Reef_site125 (AR_125) | 28 | 0.345 | 1.349 |
|  |  | Ashmore_Reef_site128 (AR_128) | 29 | 0.350 | 1.344 |
|  |  | Ashmore_Reef_site132 (AR_132) | 28 | 0.349 | 1.347 |
|  |  | Ashmore_Reef_site133 (AR_133) | 27 | 0.353 | 1.350 |
| **Rowley Shoals** | **Clerke Reef** | Rowley Shoals_Clerke Reef_C11 (RS_C_C11) | 29 | 0.348 | 1.345 |
|  |  | Rowley Shoals_Clerke Reef_C13 (RS_C_C13) | 30 | 0.348 | 1.345 |
|  |  | Rowley Shoals_Clerke Reef_C20 (RS_C_C20) | 30 | 0.347 | 1.344 |
|  |  | Rowley Shoals_Clerke Reef_S (RS_C_S) | 30 | 0.350 | 1.346 |
|  |  | Rowley Shoals_Clerke Reef_Site2 (RS_C_2) | 20 | 0.346 | 1.341 |
|  | **Imperieuse Reef** | Rowley Shoals_Imperieuse_1 (RS_I_1) | 31 | 0.352 | 1.349 |
|  |  | Rowley Shoals_Imperieuse_S (RS_I_S) | 30 | 0.350 | 1.347 |
|  | **Mermaid Reef** | Rowley Shoals_Mermaid_2 (RS_M_2) | 20 | 0.349 | 1.344 |
|  |  | Rowley Shoals_Mermaid_M11 (RS_M_M11) | 22 | 0.345 | 1.340 |
|  |  | Rowley Shoals_Mermaid_M12 (RS_M_M12) | 30 | 0.347 | 1.343 |
|  |  | Rowley Shoals_Mermaid_S (RS_M_S) | 30 | 0.350 | 1.347 |
| **inshore Kimberley** | **Adele Island** | Adele_Island_site 1 (AI_1) | 32 | 0.345 | 1.342 |
|  |  | Adele_Island_site 2 (AI_2) | 29 | 0.350 | 1.347 |
|  |  | Adele_Island_site 3 (AI_3) | 28 | 0.349 | 1.345 |
|  | **Beagle Reef** | Beagle_Reef_site_4 (BR_4) | 32 | 0.347 | 1.344 |
|  |  | Beagle_Reef_site_5 (BR_5) | 26 | 0.345 | 1.342 |
| **Ningaloo Coast World Heritage Area**  **(NWHA)** | **Ningaloo Stations** | Elle's beach site 1 (E1) | 11 | 0.328 | 1.319 |
|  |  | Elle's beach site 2 (E2) | 22 | 0.334 | 1.329 |
|  |  | Five Fingers (Coral Bay_site1) (FF1) | 5 | 0.319 | 1.299 |
|  |  | Five Fingers (Coral Bay_site2) (FF2) | 23 | 0.337 | 1.333 |
|  | **Gnaraloo** | Gnaraloo Bay Inside (GNAR) | 25 | 0.332 | 1.328 |
|  | **Quobba** | Quobba (Q) | 24 | 0.335 | 1.331 |
| **Great Barrier Reef** | **Pelorus Island** | Pelorus Island_Fat digitifera * (PF) | 12 | 0.252 | 1.245 |
|  |  | Pelorus Island_thin digitifera* (PT) | 21 | 0.270 | 1.266 |
|  | | | 704 ^a^ | 0.365^b^ | 1.334^b^ |

(*) Different morphologies were found at same site but were integrated as unique sites in genetic analyses

^a^ Total number of samples

^b^ Mean values

**Table S2.** Pairwise *F*_ST_ values between all five reef systems (including Lalang-garram Marine Park samples in the inshore Kimberley region). Bold values indicate Bonferroni adjusted significance at the 0.001 level (QC filtering: loci call rate (> 0.8) and individual call rate (> 0.3)).

|  | **Great Barrier Reef** | **Ningaloo Coast World Heritage Area** | **inshore Kimberley** | **Ashmore Reef** | **Rowley Shoals** |
| --- | --- | --- | --- | --- | --- |
| **Great Barrier Reef** | - | - | - | - | - |
| **Ningaloo Coast World Heritage Area** | **0.157** | - | - | - | - |
| **inshore Kimberley** | **0.166** | **0.145** | - | - | - |
| **Ashmore Reef** | **0.143** | **0.055** | **0.138** | - | - |
| **Rowley Shoals** | **0.150** | **0.051** | **0.158** | **0.02** | - |

**Table S3.** Pairwise *F*_ST_ values between all 14 reefs (including Lalang-garram Marine Park samples in the inshore Kimberley region). Bold values indicate Bonferroni adjusted significance at the 0.001 level (QC filtering: loci call rate (> 0.8) and individual call rate (> 0.3)).

|  | **Pelorus Island** | **Quobba** | **Gnaraloo** | **Jackson Island** | **Haywood Island** | **Ningaloo Stations** | **Augustus Island** | **Okenia Island** | **Adele Island** | **Beagle Reef** | **Ashmore Reef** | **Imperieuse Reef** | **Mermaid Reef** | **Clerke Reef** |
| --- | --- | --- | --- | --- | --- | --- | --- | --- | --- | --- | --- | --- | --- | --- |
| **Pelorus Island** | - | - | - | - | - | - | - | - | - | - | - | - | - | - |
| **Quobba** | **0.173** | - | - | - | - | - | - | - | - | - | - | - | - | - |
| **Gnaraloo** | **0.175** | -0.002 | - | - | - | - | - | - | - | - | - | - | - | - |
| **Jackson Island** | **0.773** | **0.749** | **0.751** | - | - | - | - | - | - | - | - | - | - | - |
| **Haywood Island** | **0.761** | **0.726** | **0.729** | **-0.131** | - | - | - | - | - | - | - | - | - | - |
| **Ningaloo Stations** | **0.162** | 0.000 | 0.001 | **0.731** | **0.708** | - | - | - | - | - | - | - | - | - |
| **Augustus Island** | **0.751** | **0.724** | **0.725** | -0.044 | -0.059 | **0.714** | - | - | - | - | - | - | - | - |
| **Okenia Island** | **0.715** | **0.693** | **0.696** | **0.056** | **0.196** | **0.683** | 0.003 | - | - | - | - | - | - | - |
| **Adele Island** | **0.180** | **0.092** | **0.099** | **0.735** | **0.717** | **0.089** | **0.722** | **0.691** | - | - | - | - | - | - |
| **Beagle Reef** | **0.182** | **0.097** | **0.104** | **0.741** | **0.723** | **0.093** | **0.724** | **0.694** | **0.003** | - | - | - | - | - |
| **Ashmore Reef** | **0.145** | **0.059** | **0.061** | **0.736** | **0.716** | **0.052** | **0.724** | **0.691** | **0.065** | **0.070** | - | - | - | - |
| **Imperieuse Reef** | **0.157** | **0.053** | **0.056** | **0.742** | **0.722** | **0.047** | **0.727** | **0.694** | **0.070** | **0.075** | **0.019** | - | - | - |
| **Mermaid Reef** | **0.155** | **0.054** | **0.055** | **0.737** | **0.716** | **0.047** | **0.725** | **0.692** | **0.070** | **0.076** | **0.019** | 0.000 | - | - |
| **Clerke Reef** | **0.155** | **0.055** | **0.056** | **0.736** | **0.715** | **0.049** | **0.725** | **0.692** | **0.071** | **0.077** | **0.020** | -0.001 | 0.000 | - |

**Table S4.** Correlation of environmental and geomorphological variable data, that were considered for BayeScEnv, Samßada and genetic offset models (GF and GDM), using data from WA sample site locations (based on Pearson correlation threshold of 80%). Variables in bold were retained and integrated in the models (variable abbreviations correspond to variables in Table 1)**.**

|  | Long | Lat | **SST max** | SST min | **SST range** | SST mean | SST sd | **SSTA** | TSA | **TSM** | **Chla** | **Bath** | **Rough** | **Light** | **Tidal height** |
| --- | --- | --- | --- | --- | --- | --- | --- | --- | --- | --- | --- | --- | --- | --- | --- |
| Long | 1.00 | - | - | - | - | - | - | - | - | - | - | - | - | - | - |
| Lat | 0.96 | 1.00 | - | - | - | - | - | - | - | - | - | - | - | - | - |
| **SST max** | 0.86 | 0.89 | 1.00 | - | - | - | - | - | - | - | - | - | - | - | - |
| SST min | 0.97 | 0.95 | 0.96 | 1.00 | - | - | - | - | - | - | - | - | - | - | - |
| **SST range** | -0.80 | -0.70 | -0.43 | -0.67 | 1.00 | - | - | - | - | - | - | - | - | - | - |
| SST mean | 0.92 | 0.92 | 0.99 | 0.99 | -0.56 | 1.00 | - | - | - | - | - | - | - | - | - |
| SST sd | -0.87 | -0.84 | -0.54 | -0.74 | 0.95 | -0.64 | 1.00 | - | - | - | - | - | - | - | - |
| **SSTA** | -0.83 | -0.82 | -0.69 | -0.79 | 0.69 | -0.75 | 0.77 | 1.00 | - | - | - | - | - | - | - |
| TSA | 0.98 | 0.97 | 0.79 | 0.92 | -0.83 | 0.86 | -0.93 | -0.83 | 1.00 | - | - | - | - | - | - |
| **TSM** | 0.52 | 0.50 | 0.26 | 0.38 | -0.50 | 0.33 | -0.61 | -0.54 | 0.57 | 1.00 | - | - | - | - | - |
| **Chla** | 0.14 | -0.03 | -0.17 | -0.04 | -0.29 | -0.09 | -0.25 | -0.19 | 0.12 | 0.71 | 1.00 | - | - | - | - |
| **Bath** | -0.46 | -0.44 | -0.68 | -0.59 | 0.10 | -0.64 | 0.13 | 0.42 | -0.34 | -0.14 | 0.02 | 1.00 | - | - | - |
| **Rough** | 0.05 | 0.10 | 0.28 | 0.16 | 0.20 | 0.22 | 0.17 | -0.10 | -0.01 | -0.15 | -0.24 | -0.42 | 1.00 | - | - |
| **Light** | -0.11 | -0.09 | -0.42 | -0.29 | -0.14 | -0.35 | -0.22 | -0.11 | 0.01 | 0.54 | 0.51 | 0.44 | -0.34 | 1.00 | - |
| **Tidal height** | 0.86 | 0.68 | 0.68 | 0.80 | -0.77 | 0.76 | -0.71 | -0.66 | 0.76 | 0.40 | 0.37 | -0.47 | -0.02 | -0.19 | 1.00 |

**Table S5.** Site coordinates (Lat-latitude, Lon-longitude) along with extracted environmental and geomorphological values (variable abbreviations correspond to variables in Table 1) of less correlated variables that have been used in BayeScEnv, Samßada and genetic offset model analyses.

| **Site** | **Reef** | **Lat** | **Lon** | **SST**  **max** | **SST**  **range** | **SSTA** | **TSM** | **Chla2** | **Bath** | **Rough** | **Light** | **Tidal height** |
| --- | --- | --- | --- | --- | --- | --- | --- | --- | --- | --- | --- | --- |
| **Adele Island site 1** | **Adele Island** | -15.53 | 123.20 | 31.12 | 5.51 | 0.13 | 6.58 | 4.27 | -36 | 15 | 22.26 | 2.49 |
| **Adele Island site 2** | **Adele Island** | -15.50 | 123.20 | 31.03 | 5.41 | 0.13 | 6.31 | 3.98 | -37 | 26 | 23.92 | 2.47 |
| **Adele Island site 3** | **Adele Island** | -15.55 | 123.20 | 31.20 | 5.51 | 0.13 | 5.65 | 3.81 | -4 | 11 | 21.97 | 2.53 |
| **Ashmore Reef site 125** | **Ashmore Reef** | -12.20 | 123.03 | 31.56 | 5.73 | 0.14 | 3.80 | 0.67 | -12 | 26 | 23.77 | 1.29 |
| **Ashmore Reef site 128** | **Ashmore Reef** | -12.29 | 123.12 | 31.42 | 5.59 | 0.14 | 7.18 | 2.04 | -20 | 10 | 24.10 | 1.34 |
| **Ashmore Reef site 132** | **Ashmore Reef** | -12.17 | 123.06 | 31.49 | 5.66 | 0.15 | 3.10 | 0.60 | -7 | 27 | 21.42 | 1.28 |
| **Ashmore Reef site 133** | **Ashmore Reef** | -12.27 | 122.98 | 31.57 | 5.75 | 0.13 | 9.19 | 2.72 | -18 | 25 | 22.45 | 1.31 |
| **Beagle Reef site 4** | **Beagle Reef** | -15.32 | 123.54 | 31.31 | 5.28 | 0.19 | 3.02 | 1.68 | -16 | 2 | 9.25 | 2.54 |
| **Beagle Reef site 5** | **Beagle Reef** | -15.33 | 123.54 | 31.31 | 5.29 | 0.18 | 3.12 | 1.71 | -20 | 4 | 9.28 | 2.55 |
| **Elle's beach site 1** | **Ningaloo Stations** | -23.44 | 113.78 | 27.87 | 6.58 | 0.22 | 2.08 | 1.67 | -3 | 7 | 18.05 | 0.46 |
| **Elle's beach site 2** | **Ningaloo Stations** | -23.44 | 113.78 | 27.87 | 6.58 | 0.22 | 2.09 | 1.67 | -3 | 7 | 18.01 | 0.46 |
| **Five Fingers site 1** | **Ningaloo Stations** | -23.18 | 113.76 | 28.13 | 6.62 | 0.24 | 2.34 | 1.74 | 0 | 7 | 21.30 | 0.47 |
| **Five Fingers site 2** | **Ningaloo Stations** | -23.18 | 113.76 | 28.13 | 6.62 | 0.24 | 2.34 | 1.74 | 0 | 7 | 21.30 | 0.47 |
| **Gnaraloo Bay Inside** | **Gnaraloo** | -23.82 | 113.52 | 27.87 | 6.78 | 0.17 | 0.67 | 0.99 | -10 | 22 | 20.44 | 0.46 |
| **Quobba** | **Quobba** | -24.49 | 113.41 | 27.45 | 7.42 | 0.25 | 5.57 | 4.05 | -1 | 16 | 28.19 | 0.46 |
| **Rowley Shoals Clerke Reef C11** | **Clerke Reef** | -17.35 | 119.35 | 31.45 | 6.68 | 0.18 | 3.74 | 1.54 | -32 | 3 | 16.15 | 1.32 |
| **Rowley Shoals Clerke Reef C13** | **Clerke Reef** | -17.31 | 119.37 | 31.43 | 6.71 | 0.18 | 2.90 | 1.21 | -23 | 9 | 16.01 | 1.31 |
| **Rowley Shoals Clerke Reef C20** | **Clerke Reef** | -17.31 | 119.37 | 31.43 | 6.71 | 0.18 | 2.89 | 1.21 | -19 | 9 | 16.13 | 1.31 |
| **Rowley Shoals Clerke Reef S** | **Clerke Reef** | -17.38 | 119.38 | 31.42 | 6.61 | 0.18 | 2.92 | 1.18 | -21 | 110 | 15.70 | 1.33 |
| **Rowley Shoals Clerke Reef Site 2** | **Clerke Reef** | -17.29 | 119.38 | 31.41 | 6.68 | 0.19 | 2.48 | 1.05 | -21 | 23 | 16.11 | 1.31 |
| **Rowley Shoals Imperieuse 1** | **Imperieuse Reef** | -17.55 | 118.97 | 31.36 | 6.62 | 0.20 | 3.30 | 1.19 | -40 | 7 | 13.35 | 1.25 |
| **Site** | **Reef** | **Lat** | **Lon** | **SST**  **max** | **SST**  **range** | **SSTA** | **TSM** | **Chla2** | **Bath** | **Rough** | **Light** | **Tidal height** |
| **Rowley Shoals Imperieuse S** | **Imperieuse Reef** | -17.64 | 118.96 | 31.34 | 6.60 | 0.18 | 3.77 | 1.41 | -38 | 121 | 13.90 | 1.28 |
| **Rowley Shoals Mermaid 2** | **Mermaid Reef** | -17.07 | 119.65 | 31.52 | 6.60 | 0.18 | 2.19 | 1.17 | -23 | 66 | 6.14 | 1.32 |
| **Rowley Shoals Mermaid M11** | **Mermaid Reef** | -17.13 | 119.63 | 31.53 | 6.62 | 0.18 | 2.40 | 1.32 | -30 | 28 | 6.24 | 1.33 |
| **Rowley Shoals Mermaid M12** | **Mermaid Reef** | -17.11 | 119.63 | 31.51 | 6.63 | 0.18 | 2.44 | 1.32 | -20 | 21 | 6.30 | 1.33 |
| **Rowley Shoals Mermaid S** | **Mermaid Reef** | -17.16 | 119.65 | 31.56 | 6.60 | 0.18 | 1.50 | 0.95 | -38 | 119 | 5.90 | 1.34 |

**Table S6.** Analysis of Molecular Variance (AMOVA) differentiation among reef systems, reefs, sites and samples (without Lalang-garram Marine Park samples). Degree of freedom (Df), sum of total variance across all observations (sum Sq), variance (σ) and percentage of contribution of the hierarchical component (%).

| **Hierarchical component** | **Df** | **Sum Sq** | **σ** | **%** |
| --- | --- | --- | --- | --- |
| **Between Reef system** | 4 | 9808.54 | 9.00 | **6.16** |
| **Between Reefs within Reef system** | 5 | 855.58 | -0.031 | -0.021 |
| **Between Sites within Reefs** | 18 | 3133.76 | 0.276 | 0.189 |
| **Between Samples within Sites** | 676 | 108445.3 | 23.436 | 16.026 |
| **Within samples** | 704.00 | 79939.56 | 113.551 | 77.65 |
| **Total** | 1407.00 | 202182.73 | 146.23 | 100 |

**Table S7.** Pairwise *F*_ST_ values between five reef systems (without Lalang-garram Marine Park samples). Bold values indicate Bonferroni adjusted significance at the 0.001 level (QC filtering: loci and individual call rate > 0.7).

|  | **Great Barrier Reef** | **Ningaloo Coast World Heritage Area** | **inshore Kimberley** | **Ashmore Reef** | **Rowley Shoals** |
| --- | --- | --- | --- | --- | --- |
| **Great Barrier Reef** | - | - | - | - | - |
| **Ningaloo Coast World Heritage Area** | **0.169** | - | - | - | - |
| **inshore Kimberley** | **0.167** | **0.085** | - | - | - |
| **Ashmore Reef** | **0.139** | **0.052** | **0.055** | - | - |
| **Rowley Shoals** | **0.144** | **0.045** | **0.058** | **0.02** | - |

**Table S8.** Pairwise *F*_ST_ values between all reefs (without Lalang-garram Marine Park samples). Bold values indicate Bonferroni adjusted significance at the 0.001 level (QC filtering: loci and individual call rate > 0.7).

|  | **Pelorus Island** | **Quobba** | **Gnaraloo** | **Ningaloo Stations** | **Adele Island** | **Beagle Reef** | **Ashmore Reef** | **Imperieuse Reef** | **Mermaid Reef** | **Clerke Reef** |
| --- | --- | --- | --- | --- | --- | --- | --- | --- | --- | --- |
| **Pelorus Island** | - | - | - | - | - | - | - | - | - | - |
| **Quobba** | **0.184** | - | - | - | - | - | - | - | - | - |
| **Gnaraloo** | **0.186** | 0.001 | - | - | - | - | - | - | - | - |
| **Ningaloo Stations** | **0.170** | 0.002 | 0.003 | - | - | - | - | - | - | - |
| **Adele Island** | **0.171** | **0.080** | **0.091** | **0.082** | - | - | - | - | - | - |
| **Beagle Reef** | **0.173** | **0.085** | **0.096** | **0.087** | **0.003** | - | - | - | - | - |
| **Ashmore Reef** | **0.139** | **0.053** | **0.057** | **0.050** | **0.054** | **0.058** | - | - | - | - |
| **Imperieuse Reef** | **0.150** | **0.046** | **0.049** | **0.043** | **0.057** | **0.062** | **0.017** | - | - | - |
| **Mermaid Reef** | **0.147** | **0.048** | **0.052** | **0.044** | **0.058** | **0.062** | **0.017** | 0.000 | - | - |
| **Clerke Reef** | **0.147** | **0.046** | **0.051** | **0.044** | **0.057** | **0.061** | **0.018** | -0.000 | 0.000 | - |

|  | **PT** | **Q** | **PF** | **GNAR** | **FF2** | **FF1** | **E1** | **E2** | **AI_1** | **AI_3** | **AI_2** | **BR_4** | **AR_125** | **AR_128** | **BR_5** | **RS_I_S** | **AR_132** | **AR_133** | **RS_M_2** | **RS_M_M11** | **RS_M_S** | **RS_C_2** | **RS_M_M12** | **RS_C_C20** | **RS_C_C11** | **RS_C_C13** | **RS_C_S** | **RS_I_1** |
| --- | --- | --- | --- | --- | --- | --- | --- | --- | --- | --- | --- | --- | --- | --- | --- | --- | --- | --- | --- | --- | --- | --- | --- | --- | --- | --- | --- | --- |
| **PT** | **-** | **-** | **-** | **-** | **-** | **-** | **-** | **-** | **-** | **-** | **-** | **-** | **-** | **-** | **-** | **-** | **-** | **-** | **-** | **-** | **-** | **-** | **-** | **-** | **-** | **-** | **-** | **-** |
| **Q** | **0.18** | **-** | **-** | **-** | **-** | **-** | **-** | **-** | **-** | **-** | **-** | **-** | **-** | **-** | **-** | **-** | **-** | **-** | **-** | **-** | **-** | **-** | **-** | **-** | **-** | **-** | **-** | **-** |
| **PF** | **0.06** | **0.19** | **-** | **-** | **-** | **-** | **-** | **-** | **-** | **-** | **-** | **-** | **-** | **-** | **-** | **-** | **-** | **-** | **-** | **-** | **-** | **-** | **-** | **-** | **-** | **-** | **-** | **-** |
| **GNAR** | **0.19** | 0.00 | **0.20** | **-** | **-** | **-** | **-** | **-** | **-** | **-** | **-** | **-** | **-** | **-** | **-** | **-** | **-** | **-** | **-** | **-** | **-** | **-** | **-** | **-** | **-** | **-** | **-** | **-** |
| **FF2** | **0.17** | 0.00 | **0.19** | 0.00 | - | - | **-** | **-** | **-** | **-** | **-** | **-** | **-** | **-** | **-** | **-** | **-** | **-** | **-** | **-** | **-** | **-** | **-** | **-** | **-** | **-** | **-** | **-** |
| **FF1** | **0.19** | 0.00 | **0.21** | 0.00 | 0.00 | - | **-** | **-** | **-** | **-** | **-** | **-** | **-** | **-** | **-** | **-** | **-** | **-** | **-** | **-** | **-** | **-** | **-** | **-** | **-** | **-** | **-** | **-** |
| **E1** | **0.18** | 0.00 | **0.20** | 0.00 | 0.00 | -0.01 | **-** | **-** | **-** | **-** | **-** | **-** | **-** | **-** | **-** | **-** | **-** | **-** | **-** | **-** | **-** | **-** | **-** | **-** | **-** | **-** | **-** | **-** |
| **E2** | **0.18** | 0.00 | **0.19** | 0.00 | 0.00 | 0.00 | 0.00 | **-** | **-** | **-** | **-** | **-** | **-** | **-** | **-** | **-** | **-** | **-** | **-** | **-** | **-** | **-** | **-** | **-** | **-** | **-** | **-** | **-** |
| **AI_1** | **0.18** | **0.08** | **0.19** | **0.10** | **0.09** | **0.08** | **0.08** | **0.09** | **-** | **-** | **-** | **-** | **-** | **-** | **-** | **-** | **-** | **-** | **-** | **-** | **-** | **-** | **-** | **-** | **-** | **-** | **-** | **-** |
| **AI_3** | **0.18** | **0.08** | **0.19** | **0.09** | **0.08** | **0.07** | **0.08** | **0.08** | 0.00 | - | - | **-** | **-** | **-** | **-** | **-** | **-** | **-** | **-** | **-** | **-** | **-** | **-** | **-** | **-** | **-** | **-** | **-** |
| **AI_2** | **0.18** | **0.08** | **0.19** | **0.09** | **0.08** | **0.07** | **0.08** | **0.08** | 0.00 | 0.00 | - | **-** | **-** | **-** | **-** | **-** | **-** | **-** | **-** | **-** | **-** | **-** | **-** | **-** | **-** | **-** | **-** | **-** |
| **BR_4** | **0.17** | **0.08** | **0.19** | **0.10** | **0.09** | **0.08** | **0.08** | **0.09** | 0.00 | 0.00 | 0.00 | **-** | **-** | **-** | **-** | **-** | **-** | **-** | **-** | **-** | **-** | **-** | **-** | **-** | **-** | **-** | **-** | **-** |
| **AR_125** | **0.14** | **0.06** | **0.16** | **0.06** | **0.05** | **0.05** | **0.05** | **0.06** | **0.06** | **0.05** | **0.05** | **0.06** | **-** | **-** | **-** | **-** | **-** | **-** | **-** | **-** | **-** | **-** | **-** | **-** | **-** | **-** | **-** | **-** |
| **AR_128** | **0.14** | **0.05** | **0.16** | **0.06** | **0.05** | **0.04** | **0.04** | **0.05** | **0.06** | **0.05** | **0.05** | **0.06** | 0.00 | **-** | **-** | **-** | **-** | **-** | **-** | **-** | **-** | **-** | **-** | **-** | **-** | **-** | **-** | **-** |
| **BR_5** | **0.18** | **0.09** | **0.19** | **0.10** | **0.09** | **0.09** | **0.08** | **0.09** | 0.00 | 0.00 | 0.00 | 0.00 | **0.06** | **0.06** | **-** | **-** | **-** | **-** | **-** | **-** | **-** | **-** | **-** | **-** | **-** | **-** | **-** | **-** |
| **RS_I_S** | **0.15** | **0.05** | **0.17** | **0.05** | **0.04** | **0.04** | **0.04** | **0.04** | **0.06** | **0.05** | **0.05** | **0.06** | **0.02** | **0.01** | **0.06** | **-** | **-** | **-** | **-** | **-** | **-** | **-** | **-** | **-** | **-** | **-** | **-** | **-** |
| **AR_132** | **0.15** | **0.06** | **0.16** | **0.06** | **0.05** | **0.04** | **0.05** | **0.06** | **0.06** | **0.06** | **0.05** | **0.06** | 0.00 | 0.00 | **0.06** | **0.02** | **-** | **-** | **-** | **-** | **-** | **-** | **-** | **-** | **-** | **-** | **-** | **-** |
| **AR_133** | **0.15** | **0.05** | **0.16** | **0.06** | **0.05** | **0.05** | **0.04** | **0.05** | **0.06** | **0.05** | **0.05** | **0.06** | 0.00 | 0.00 | **0.06** | **0.01** | 0.00 | **-** | **-** | **-** | **-** | **-** | **-** | **-** | **-** | **-** | **-** | **-** |
| **RS_M_2** | **0.15** | **0.05** | **0.17** | **0.05** | **0.04** | **0.04** | **0.04** | **0.04** | **0.06** | **0.06** | **0.06** | **0.06** | **0.02** | **0.02** | **0.06** | 0.00 | **0.02** | **0.02** | **-** | **-** | **-** | **-** | **-** | **-** | **-** | **-** | **-** | **-** |
| **RS_M_M11** | **0.16** | **0.05** | **0.17** | **0.06** | **0.05** | **0.04** | **0.04** | **0.05** | **0.07** | **0.06** | **0.06** | **0.07** | **0.02** | **0.02** | **0.07** | 0.00 | **0.02** | **0.02** | 0.01 | - | - | - | - | - | - | - | - | - |
| **RS_M_S** | **0.16** | **0.05** | **0.17** | **0.05** | **0.04** | **0.04** | **0.04** | **0.05** | **0.06** | **0.06** | **0.05** | **0.06** | **0.02** | **0.02** | **0.06** | 0.00 | **0.02** | **0.02** | 0.00 | 0.00 | - | - | - | - | - | - | - | - |
| **RS_C_2** | **0.16** | **0.05** | **0.18** | **0.06** | **0.05** | **0.04** | **0.04** | **0.05** | **0.06** | **0.06** | **0.05** | **0.06** | **0.02** | **0.02** | **0.07** | 0.00 | **0.02** | **0.02** | 0.00 | 0.01 | 0.00 | - | - | - | - | - | - | - |
| **RS_M_M12** | **0.15** | **0.05** | **0.17** | **0.06** | **0.05** | **0.04** | **0.04** | **0.05** | **0.06** | **0.06** | **0.05** | **0.06** | **0.02** | **0.02** | **0.06** | 0.00 | **0.02** | **0.02** | 0.00 | 0.00 | 0.00 | 0.00 | - | - | - | - | - | - |
| **RS_C_C20** | **0.15** | **0.05** | **0.17** | **0.05** | **0.04** | **0.04** | **0.04** | **0.04** | **0.06** | **0.06** | **0.05** | **0.06** | **0.02** | **0.02** | **0.06** | 0.00 | **0.02** | **0.02** | 0.00 | 0.00 | 0.00 | 0.00 | 0.00 | - | - | - | - | - |
| **RS_C_C11** | **0.16** | **0.05** | **0.17** | **0.05** | **0.04** | **0.04** | **0.04** | **0.05** | **0.06** | **0.06** | **0.05** | **0.06** | **0.02** | **0.02** | **0.06** | 0.00 | **0.02** | **0.02** | 0.00 | 0.00 | 0.00 | 0.00 | 0.00 | 0.00 | - | - | - | - |
| **RS_C_C13** | **0.15** | **0.05** | **0.17** | **0.05** | **0.05** | **0.04** | **0.04** | **0.04** | **0.06** | **0.06** | **0.05** | **0.06** | **0.02** | **0.02** | **0.06** | 0.00 | **0.02** | **0.02** | 0.00 | 0.00 | 0.00 | 0.00 | 0.00 | 0.00 | 0.00 | - | - | - |
| **RS_C_S** | **0.15** | **0.05** | **0.17** | **0.05** | **0.05** | **0.04** | **0.04** | **0.05** | **0.06** | **0.06** | **0.06** | **0.06** | **0.02** | **0.01** | **0.06** | 0.00 | **0.02** | **0.02** | 0.00 | 0.00 | 0.00 | 0.00 | 0.00 | 0.00 | 0.00 | 0.00 | - | - |
| **RS_I_1** | **0.15** | **0.05** | **0.16** | **0.05** | **0.04** | **0.04** | **0.04** | **0.05** | **0.06** | **0.06** | **0.06** | **0.06** | **0.02** | **0.02** | **0.06** | 0.00 | **0.02** | **0.02** | 0.00 | 0.00 | 0.00 | 0.00 | 0.00 | 0.00 | 0.00 | 0.00 | 0.00 | - |

**Table S9.** Pairwise *F*_ST_ values between all 28 sites (without Lalang-garram Marine Park samples). Bold values indicate Bonferroni adjusted significance at the 0.001 level. Abbreviations corresponding to sites can be found in Table S1.

**Table S10.** Pairwise *F*_ST_ values between four reef systems (without Lalang-garram Marine Park and Great Barrier Reef samples). Bold values indicate Bonferroni adjusted significance at the 0.001 level (QC filtering: loci and individual call rate > 0.7).

|  | **Ningaloo Coast World Heritage Area** | **inshore Kimberley** | **Ashmore Reef** | **Rowley Shoals** |
| --- | --- | --- | --- | --- |
| **Ningaloo Coast World Heritage Area** | - | - | - | - |
| **inshore Kimberley** | **0.081** | - | - | - |
| **Ashmore Reef** | **0.051** | **0.055** | - | - |
| **Rowley Shoals** | **0.046** | **0.057** | **0.02** | - |

**Table S11.** Analysis of Molecular Variance (AMOVA) differentiation among reef systems, reefs, sites and samples in WA (without Lalang-garram Marine Park and Great Barrier Reef samples). Degree of freedom (Df), sum of total variance across all observations (sum Sq), variance (σ) and percentage of contribution of the hierarchical component (%).

| **Hierarchical component** | **Df** | **Sum Sq** | **σ** | **%** |
| --- | --- | --- | --- | --- |
| **Between Reef system** | 3.00 | 7511.70 | 7.45 | **4.76** |
| **Between Reefs within Reef system** | 5.00 | 928.97 | 0.04 | 0.02 |
| **Between Sites within Reefs** | 17.00 | 3071.60 | 0.09 | 0.06 |
| **Between Samples within Sites** | 645.00 | 113585.43 | 27.20 | 17.38 |
| **Within samples** | 671.00 | 81659.04 | 121.70 | 77.77 |
| **Total** | 1341.00 | 206756.74 | 156.48 | 100.00 |

**Table S12.** Samβada output of significant gene-environment associations (GEAs) after Bonferroni correction and highest G score filtering for each genotype-environmental combination. Reef systems in WA are classified according to their highest population membership coefficient in each population (pop 1, pop 2 and pop 3). Numbers correspond to number of significant GEA linked to top three most important drivers of outlier genotype structure: tidal height, SST range and SST max.

| **Locations** | **Population membership** | **SSTrange** | **Tidal height** | **SSTmax** |
| --- | --- | --- | --- | --- |
| **Ningaloo Coast World Heritage Area** | pop1, pop3 | 160 | 87 | 60 |
| **Rowley Shoals** | pop1, pop2 | 125 | 170 | 76 |
| **inshore Kimberley** | pop1, pop 2, pop3 | 232 | 226 | 101 |
| **Ashmore Reef** | pop2, pop3 | 179 | 195 | 66 |

**Table S13.** Genetic offset predictions among reef systems in WA for each future climate scenarios using gradient forest (GF) and generalised dissimilarity models (GDM). Values represent minimum (min), maximum (max), median, standard deviation (std) and 95
% confidence intervals (CI_lower/CI_upper) Euclidean distance values between present and future GEAs.

| Reef system | Scenario | Model | Min | Max | Mean | Median | std | CI_lower | CI_upper |
| --- | --- | --- | --- | --- | --- | --- | --- | --- | --- |
| Ashmore Reef | RCP2.6 (2050) | GF | 0.045 | 0.099 | 0.071 | 0.073 | 0.021 | 0.070 | 0.071 |
| Ashmore Reef | RCP2.6 (2050) | GDM | 0.098 | 0.099 | 0.099 | 0.098 | 0.000 | 0.099 | 0.099 |
| inshore Kimberley | RCP2.6 (2050) | GF | 0.002 | 0.118 | 0.036 | 0.019 | 0.031 | 0.035 | 0.036 |
| inshore Kimberley | RCP2.6 (2050) | GDM | 0.098 | 0.103 | 0.099 | 0.098 | 0.001 | 0.099 | 0.099 |
| Rowley Shoals | RCP2.6 (2050) | GF | 0.104 | 0.116 | 0.113 | 0.113 | 0.003 | 0.113 | 0.113 |
| Rowley Shoals | RCP2.6 (2050) | GDM | 0.106 | 0.110 | 0.108 | 0.107 | 0.001 | 0.107 | 0.108 |
| Ningaloo World Heritage Area | RCP2.6 (2050) | GF | 0.026 | 0.146 | 0.098 | 0.098 | 0.020 | 0.097 | 0.098 |
| Ningaloo World Heritage Area | RCP2.6 (2050) | GDM | 0.098 | 0.357 | 0.194 | 0.169 | 0.083 | 0.193 | 0.195 |
| Ashmore Reef | RCP2.6 (2100) | GF | 0.042 | 0.096 | 0.068 | 0.069 | 0.020 | 0.068 | 0.068 |
| Ashmore Reef | RCP2.6 (2100) | GDM | 0.098 | 0.099 | 0.099 | 0.098 | 0.000 | 0.099 | 0.099 |
| inshore Kimberley | RCP2.6 (2100) | GF | 0.005 | 0.122 | 0.040 | 0.026 | 0.035 | 0.040 | 0.040 |
| inshore Kimberley | RCP2.6 (2100) | GDM | 0.098 | 0.103 | 0.099 | 0.099 | 0.001 | 0.099 | 0.099 |
| Rowley Shoals | RCP2.6 (2100) | GF | 0.100 | 0.114 | 0.110 | 0.110 | 0.003 | 0.109 | 0.110 |
| Rowley Shoals | RCP2.6 (2100) | GDM | 0.109 | 0.113 | 0.110 | 0.110 | 0.001 | 0.110 | 0.111 |
| Ningaloo World Heritage Area | RCP2.6 (2100) | GF | 0.021 | 0.130 | 0.082 | 0.085 | 0.027 | 0.082 | 0.082 |
| Ningaloo World Heritage Area | RCP2.6 (2100) | GDM | 0.098 | 0.382 | 0.237 | 0.221 | 0.075 | 0.237 | 0.238 |
| Ashmore Reef | RCP8.5 (2050) | GF | 0.045 | 0.098 | 0.070 | 0.070 | 0.020 | 0.069 | 0.070 |
| Ashmore Reef | RCP8.5 (2050) | GDM | 0.098 | 0.099 | 0.099 | 0.098 | 0.000 | 0.099 | 0.099 |
| inshore Kimberley | RCP8.5 (2050) | GF | 0.016 | 0.120 | 0.045 | 0.027 | 0.030 | 0.044 | 0.045 |
| inshore Kimberley | RCP8.5 (2050) | GDM | 0.098 | 0.103 | 0.099 | 0.098 | 0.001 | 0.099 | 0.099 |
| Reef system | **Scenario** | **Model** | **Min** | **Max** | **Mean** | **Median** | **std** | **CI_lower** | **CI_upper** |
| Rowley Shoals | RCP8.5 (2050) | GF | 0.105 | 0.119 | 0.115 | 0.115 | 0.003 | 0.115 | 0.115 |
| Rowley Shoals | RCP8.5 (2050) | GDM | 0.099 | 0.101 | 0.100 | 0.100 | 0.000 | 0.100 | 0.100 |
| Ningaloo World Heritage Area | RCP8.5 (2050) | GF | 0.021 | 0.123 | 0.080 | 0.081 | 0.026 | 0.080 | 0.081 |
| Ningaloo World Heritage Area | RCP8.5 (2050) | GDM | 0.099 | 0.387 | 0.258 | 0.251 | 0.064 | 0.257 | 0.258 |
| Ashmore Reef | RCP8.5 (2100) | GF | 0.081 | 0.119 | 0.099 | 0.099 | 0.015 | 0.099 | 0.099 |
| Ashmore Reef | RCP8.5 (2100) | GDM | 0.098 | 0.099 | 0.099 | 0.098 | 0.000 | 0.099 | 0.099 |
| inshore Kimberley | RCP8.5 (2100) | GF | 0.071 | 0.140 | 0.094 | 0.093 | 0.018 | 0.093 | 0.094 |
| inshore Kimberley | RCP8.5 (2100) | GDM | 0.098 | 0.103 | 0.099 | 0.098 | 0.001 | 0.099 | 0.099 |
| Rowley Shoals | RCP8.5 (2100) | GF | 0.115 | 0.128 | 0.124 | 0.124 | 0.003 | 0.124 | 0.124 |
| Rowley Shoals | RCP8.5 (2100) | GDM | 0.098 | 0.099 | 0.098 | 0.098 | 0.000 | 0.098 | 0.098 |
| Ningaloo World Heritage Area | RCP8.5 (2100) | GF | 0.091 | 0.153 | 0.132 | 0.134 | 0.006 | 0.132 | 0.132 |
| Ningaloo World Heritage Area | RCP8.5 (2100) | GDM | 0.257 | 0.449 | 0.382 | 0.389 | 0.040 | 0.382 | 0.383 |

**Table S14.** Mantel correlation test values, evaluating the correlation between geographic and environmental distance matrix. Mantel statistic r shows the correlation value a specified significance level.

| **Environmental variable** | **Mantel statistic r** | **Significance level** |
| --- | --- | --- |
| SST max | 0.74414 | < 0.001 |
| SST anomalies (SSTA) | 0.649 | < 0.001 |
| Tidal height | 0.48 | < 0.001 |
| SST range | 0.4036 | < 0.001 |
| bath | 0.3866 | < 0.001 |
| TSM | 0.2214 | < 0.05 |
| Chlorophyll a | 0.2178 | < 0.05 |
| Light | 0.1535 | < 0.05 |
| Rough | -0.08696 | > 0.05 |
| **All variables** | **0.05566** | **> 0.05** |

**Supplementary Figures**

**Figure S1**. DAPC clustering of all 14 reefs in WA and Great Barrier Reef (Pelorus Island) using SNP genotype data, filtered with loci call rate (> 0.8) and individual call rate (> 0.3) parameters. Haywood Island, Jackson Island Augustus Island and Okenia Island are part of the Lalang-garram Marine Park in the inshore Kimberley reef system.

**Figure S2**. Quality score filtering plots of DArT SNP genotype data. A) Pre- and post-filtering plots by call rate for loci and (B) individuals. After QC, Heterozygosity (C) and *F*_ST_ plots (D) for *Acropora digitifera* genotype data were constructed. Red points represent loci identified by BayeScan as outliers and were separated from further analyses.

1.
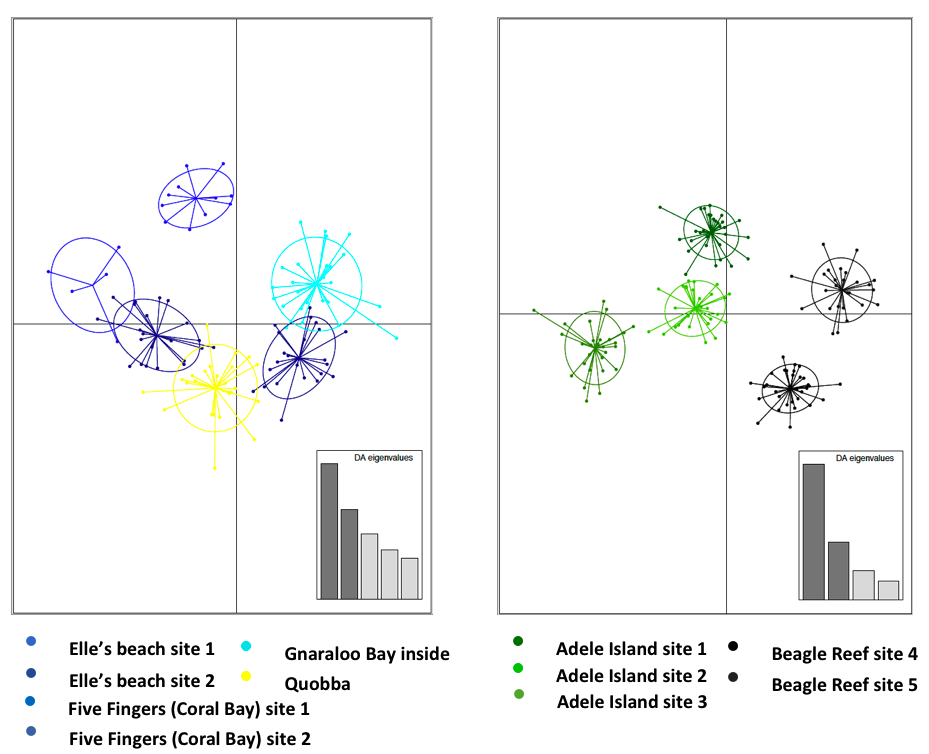

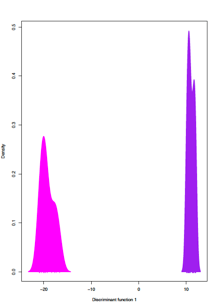

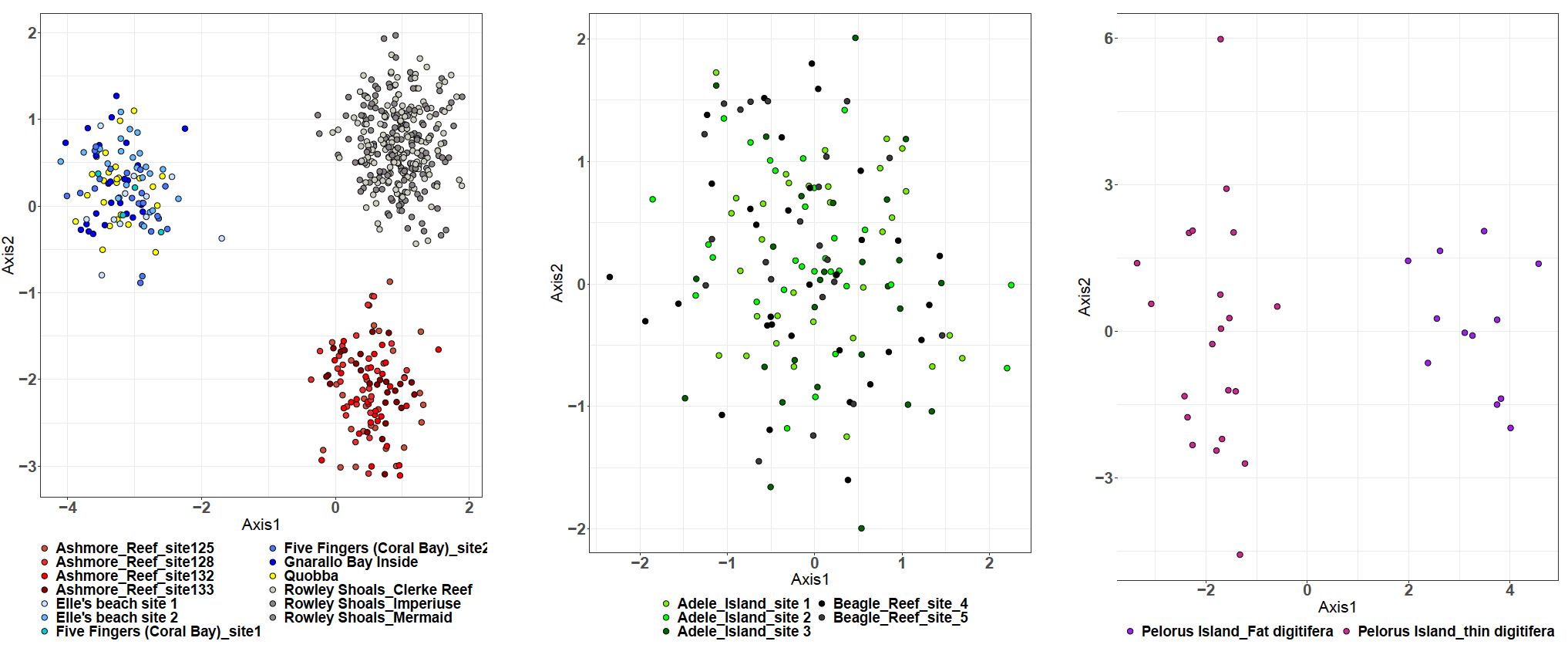
 **B) C)**

**Figure S3.** Fine spatial scale DAPC plots. Low genetic differentiation was detected across sites within Ningaloo World Heritage Area (A) while Adele Island and Beagle Reef sites show distinct genetic structure (B). Distinct clustering was found in DAPC (top right) and PCA plot between thin and fat branching *A. digitifera* morphologies at Pelorus Island reef (GBR) (C).

**Figure S4.** Variable importance of GF model algorithm, constructed by integrating minor allele frequencies of 38 outlier loci with extracted environmental and habitat variables at every site. Tidal height showed to be most important variable explaining GEAs at WA sites followed by SSTmax, SSTrange, Bathymetry and SSTA.

**Figure S5.** Cumulative variable importance of environmental and geomorphological variables in the final GF model, explaining the genetic signature across the sampled sites in WA for each of the 38 individual outlier loci with positive weighted R^2^ goodness of fit.

**Figure S6.** Relative variable importance of GDM, fitted by I-splines, with tidal height, SSTmax, geographic distance as only significant contributing variables,

**Figure S7.** PCA showing the similarity in gene-environmental associations within the 50 km buffer zone of the 26 sampled sites in WA, (red, green and blue are assigned using PC1, PC2 and PC3 combinations) using the best GDM. In this plot, the more similar the colours, the more similar areas, that neighbour sampled sites, are in terms of genetic composition with those sample sites. Vectors represent the direction and magnitude of the five most explanatory variables (SSTrange, Tidal height, SSTmax, SSTA and Bathymetry in decreasing order). Small black circles represent site locations encircled by reefs. From left to right (green – Adele Island, black – Beagle Reef (inshore Kimberley), red – Ashmore Reef, grey – Imperieuse, Clerke and Mermaid Reef (Rowley Shoals), dark blue – Ningaloo Stations, magenta – Gnaraloo, yellow – Quobba (Ningaloo Coast World Heritage Area) (left panel). Notched boxplots representing the variability in the genetic offset, represented by the Euclidean distance between present-day and future genetic space, across reef systems in WA under RCP 2.6 and RCP 8.5 in 2040-2050 and 2090-2100. Red circles represent mean values while black dots represent outliers (right panel).


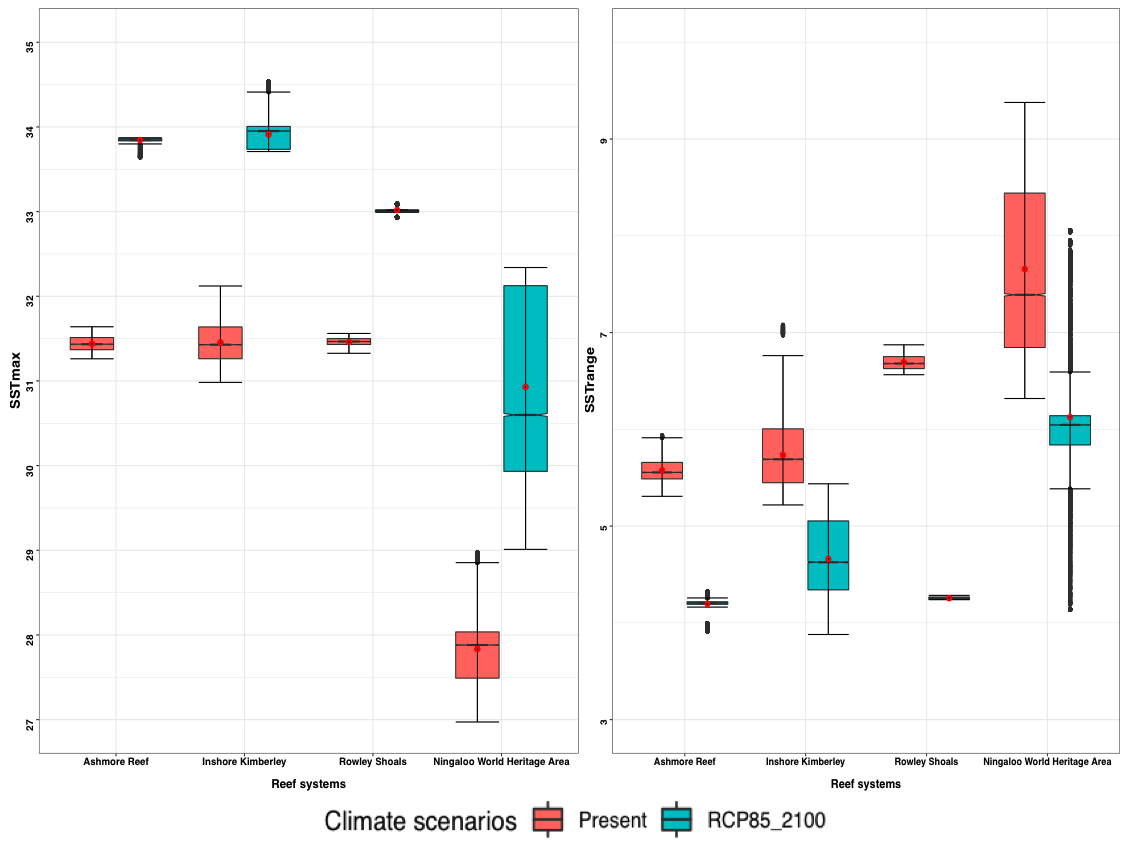


**Figure S8.** Regional variability in present-day and future (RCP 8.5 in 2090-2100) SSTmax and SSTrange data across the reef systems in WA; Ashmore Reef, inshore Kimberley, Rowley Shoals and Ningaloo Coast World Heritage Area.

**Figure S9.** Regression plots showing top three highest significant correlations between geographic distance across sites and difference in environmental data. (Mantel; SSTmax r = 0.74, SSTA = 0.649, Tidal height = 0.48, Table S14), as well as correlation of distance with all variable data (r = 0.056, *P* > 0.05).

**Figure S10.** Samβada population clustering plots. A) wss (within sum of squares) plot shows the optimal number of populations (K = 4). B) Number of PCs corresponding to the proportion of explained variance of population structure (PC = 3) was selected as optimal number of PCs to segregate neutral loci population structure in Samβada with adequate portion of explained variance.

**References**

Duruz, S., Sevane, N., Selmoni, O., Vajana, E., Leempoel, K., Stucki, S., . . . Joost, S. (2019). Rapid identification and interpretation of gene–environment associations using the new R.SamBada landscape genomics pipeline. *Molecular Ecology Resources,* 19(5), 1355-1365.

Selmoni, O., Rochat, E., Lecellier, G., Berteaux‐Lecellier, V., & Joost, S. (2020). Seascape genomics as a new tool to empower coral reef conservation strategies: An example on north‐western Pacific *Acropora digitifera*. *Evolutionary Applications,* 13(8), 1923-1938.

Stucki, S., Orozco-terWengel, P., Forester, B. R., Duruz, S., Colli, L., Masembe, C., . . . Joost, S. (2017). High performance computation of landscape genomic models including local indicators of spatial association. *Molecular Ecology Resources,* 17(5), 1072-1089.
